# Bilirubin reduces mortality in sepsis models by inhibiting NOX2-mediated formation of neutrophil extracellular traps

**DOI:** 10.1080/13510002.2026.2664962

**Published:** 2026-04-28

**Authors:** Sang-Jin Kim, Chang Woo Ko, Wan-Seo Kim, Yeon Jun Kang, Chul-Hwan Lee, Won-Woo Lee, Jong-Wan Park

**Affiliations:** a Cancer Research Institute and Ischemic/Hypoxic Disease Institute, Seoul National University College of Medicine, Seoul, Republic of Korea; b Department of Pharmacology, Seoul National University College of Medicine, Seoul, Republic of Korea; c Department of Biomedical Sciences, Seoul National University College of Medicine, Seoul, Republic of Korea; d Department of Microbiology and Immunology, Seoul National University College of Medicine, Seoul, Republic of Korea

**Keywords:** Sepsis, bilirubin, neutrophil extracellular traps, NADPH oxidase

## Abstract

**Objectives:**

Sepsis is a life-threatening condition driven by a dysregulated immune response to infection, yet therapeutic options beyond antibiotics and vasopressors remain limited. Neutrophil extracellular traps (NETs) contribute significantly to sepsis-induced tissue injury, and NETosis inhibition has emerged as a potential therapeutic strategy. We hypothesized that the endogenous metabolite bilirubin mitigates inflammation in sepsis by inhibiting NETosis through targeting NOX2.

**Methods:**

Two murine sepsis models were used to assess the effects of bilirubin on survival and systemic NETosis. Plasma NET biomarkers were quantified, and primary human neutrophils were used to validate the NETosis-inhibitory activity of bilirubin *in vitro*. Mechanistic studies included ROS measurements, NOX2 loop C mutational analysis, and inhibition of endocytosis and autophagy to examine how bilirubin modulates NOX2 stability.

**Results:**

Bilirubin improved survival and reduced NET biomarkers in both models. It inhibited NETosis in human neutrophils by suppressing ROS-dependent NETosis and promoting the internalization and degradation of NOX2 via endocytosis and autophagy.

**Discussion:**

These findings identify bilirubin as an endogenous inhibitor of NETosis. By targeting NOX2 and suppressing NETosis, bilirubin may represent a promising therapeutic candidate for sepsis management.

## Graphical abstract

**Figure:**
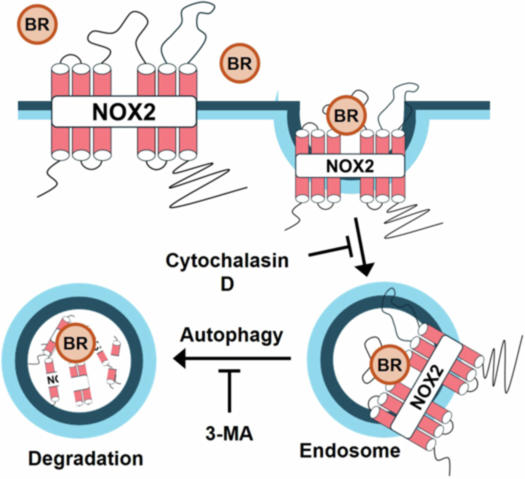

## 1. Introduction

Sepsis is a dysregulated immune response to infection, leading to systemic inflammation, tissue damage, and multiple organ failure [1,2]. Despite advances in medical care, sepsis is still one of the leading causes of death and disability around the world. A growing body of evidence indicates that neutrophil extracellular traps (NETs) play a crucial role in tissue injury during sepsis [3–5]. NETs are composed of chromatin structures decorated with antimicrobial proteins, which have beneficial effects to eliminate pathogens [6,7]. However, excessive NETs can cause tissue damage and exacerbate inflammation, thus playing a detrimental role in the progression of sepsis [8,9]. Indeed, NET components, such as histones and proteases, have been reported to damage surrounding tissues by breaking down cellular structures and extracellular matrices [10–12]. Additionally, excessive NETs can trigger a sustained inflammatory response by activating immune cells to release pro-inflammatory cytokines, leading to the secondary events of tissue injury, organ dysfunction and further exacerbation of septic symptoms [8,13,14].

NADPH oxidase 2 (NOX2) is the key enzyme that generates reactive oxygen species (ROS), including superoxide anion (O_2_•-) in activated neutrophils [15]. While ROS are essential for pathogen clearance, their overproduction during sepsis can lead to oxidative injuries to surrounding tissues [16,17]. NOX2 also plays a crucial role in NETosis by generating ROS, which induces the release of neutrophil enzymes from azurophilic granules and activate peptidyl arginine deiminase 4 (PAD4) by inducing thioredoxin (TRX) [18–21]. Activated PAD4 decondenses nuclear chromatin by citrullinating histones, which is essential for NETosis [22,23]. Therefore, NOX2 is a promising target to control sepsis because NOX2 inhibitors could quench a signaling process toward NETosis.

Bilirubin is a natural product of heme catabolism and was previously regarded as a metabolic waste to be excreted out of our body [24,25]. The blood level of bilirubin ranges from 0.2 to 1.2 mg/dL in normal adults but can be highly fluctuating under some pathologic conditions. In case of neonatal jaundice, its level goes up to 10 mg/dL or more [26,27]. Also, its blood level can rise to several hundred mg/dL in patients with biliary obstruction [28]. Interestingly, the bilirubin level in sepsis patients was detected at the range of 2.5 and 20 mg/dL [29,30]. Given that the bilirubin level is relatively high and dynamically regulated in diverse situations, it is expected that bilirubin may play some biological roles beyond a metabolic waste. Recent studies have highlighted its biological roles in cell signaling pathways [31,32]. For instance, bilirubin has been shown to attenuate inflammatory processes by activating PPAR-α. Bilirubin directly binds with PPAR-α and recruits the nuclear receptor to the PPAR response elements in the promotors of the target genes, thereby increasing gene expressions [33]. In addition, bilirubin has been shown to attenuate oxidative stress and inflammation [31,32]. Bilirubin can scavenge ROS through a chemical reaction or reduce ROS production from NOX2 in a cell-free system [34–37]. These anti-inflammatory actions of bilirubin prompted us to make a hypothesis that bilirubin blocks NETosis by inhibiting inflammatory processes in neutrophils.

In the present study, we explored the possibility that bilirubin improves survival in sepsis by blocking NETosis. We employed two sepsis mouse models, a cecal ligation and puncture (CLP) model and a lipopolysaccharide (LPS) model to evaluate the *in vivo* effects of bilirubin on sepsis. These two models represent complementary aspects of sepsis pathophysiology. The LPS-induced endotoxemia model provides a controlled system to study endotoxin-driven systemic inflammatory responses. The CLP model more closely reflects polymicrobial infection and the complex host–pathogen interactions observed in clinically relevant sepsis. Additionally, we investigated the molecular mechanisms underlying bilirubin's inhibitory actions against NETosis in human neutrophils. A better understanding about the NETosis-inhibitory actions of bilirubin will provide a new insight into clinical management of patients with sepsis.

## 2. Materials and methods

### 2.1. Reagent and antibodies

Bilirubin (B4126), biliverdin (30891), phorbol 12-myristate 13-acetate (PMA, P8139), lipopolysaccharides from Escherichia coli (LPS, L2630), H_2_O_2_ (216763), N-Acetyl-L-cysteine (NAC, A750), diphenyleneiodonium chloride (DPI, D2926), GW6471 (G5045), cycloheximide (01810), MG132 (10012628), bafilomycin A1 (B1793), 3-methyladenine (189490), cytochalasin D (C2618), cytochrome C (C2037) and superoxide dismutase (S5395) were purchased from Sigma-Aldrich (St. Louis, MO); Fenofibrate (HY-17356) from MedChemExpress (Monmouth Junction, NJ); Recrystallized bilirubin (#0219947483) from MP biomedicals (Irvine, CA); NADPH (481973) from Calbiochem (Burlington, MA). Antibodies against H3 (3638, 1:5000), LC3B (2775, 1:2000), Rab7 (2094S, 1:1000) and GAPDH (2118, 1:5000) were purchased from Cell Signaling Technology (Danvers, MA); anti-NOX2 antibody (ab80897, 1:2000) from Abcam (Cambridge, UK); Anti-H3cit antibody (17939, 1:2000) from Cayman Chemical (Ann Arbor, MI); anti-FLAG antibody (F7425, 1:2000) from Sigma-Aldrich (St. Louis, MO). For *in vitro* cell-based experiments, bilirubin was first solubilized in 0.2 N NaOH and subsequently dissolved in Hank's balanced salt solution (H6648, Sigma Aldrich, St. Louis, MO) supplemented with 1% BSA, followed by neutralization to pH 7.4 with 0.5 N HCl.

### 2.2. Plasmids and transfection

The cDNA for human NOX2 (NM_000397) was cloned by RT-PCR using Pfu DNA polymerase. The cDNA was inserted into the pcDNA-FLAG plasmid via blunt-end ligation. Mutations and deletions in the cDNA were generated using PCR-based mutagenesis. For transient transfection, cells were cultured to ~70% confluence and transfected with plasmids using jetPrime reagent (Polyplus, Illkirch, France). After stabilizing for 48 h, cells were subjected to experiments. Western blotting was performed to confirm the expression of the transfected gene.

### 2.3. Preparation of a recombinant protein of NOX2 101–205

The cDNA for NOX2 101–205 was inserted into the pET28 plasmid to express the 6xHis-tagged recombinant protein from *E*. coli. The expression of the recombinant His-NOX2 101–205 protein was induced in BL21 *E*. *coli* at 12 °C for 16 h with isopropyl β-D-1-thiogalactopyranoside (1 M). Bacteria were lysed by intermittent sonication on ice (4-s interval for 5 min), and the lysates were centrifuged at 10,000 × *g* for 30 min. The soluble fraction was prepared from the lysates in the absence of urea. The His-NOX2 101–205 protein was purified using nickel-NTA beads (Qiagen, Hilden, Germany) at 4 °C for 4 h and eluted with 250 mM imidazole. The eluted proteins were concentrated 10-fold using an Amicon centrifugal filter (Merck, Darmstadt, Germany). Protein amount and purity were assessed by SDS–PAGE and EZ-gel staining (DogenBio, Seoul, South Korea).

### 2.4. Neutrophil isolation and culture

The study protocols were reviewed and approved by the Institutional Review Board of Seoul National University Hospital (Approval No. 2021-157-1184) and carried out according to the Helsinki Declaration. After obtaining written informed consent, peripheral blood from healthy volunteers was collected in accordance with the approved guidelines and regulations. EDTA-treated blood was diluted in PBS and layered onto Bicoll separating solution (BIOCHROM; Cambridge, UK). The mixture was centrifuged at 700 × *g* for 20 min. Following density gradient separation, the RBC-granulocyte pellet was resuspended in an equal volume of warm Hank's Balanced Salt Solution (H6648, Sigma Aldrich, St. Louis, MO) and centrifuged at 220 × *g* for 10 min. The supernatant was discarded, and red blood cells (RBCs) were lysed twice using a lysis buffer containing 155 mM ammonium chloride, 12 mM sodium bicarbonate, and 0.1 mM EDTA. The remaining cell pellet was resuspended in RPMI 1640 medium (WELGENE; Gyeongsan, Korea) containing 10% FBS. Neutrophils were cultured at 37 °C in a humidified atmosphere containing 5% CO₂.

### 2.5. Animal models for sepsis

Animal maintenance and experimental procedures were conducted in accordance with institutional and national guidelines for the care and use of laboratory animals (Approval No. SNU-230601-1-1). Male BALB/c mice aged 6–8 weeks (20–25 g) were obtained from Orient Bio (Seongnam, Korea). All animals were randomly allocated to each experimental group with 15 mice per group. Group allocation was known to the experimenter at all stages of the study. Mice were anesthetized with isoflurane inhalation and remained under anesthesia throughout the surgical procedures. For the cecal ligation and puncture (CLP) model to induce sepsis, the cecum was ligated approximately 70% using 4-0 silk sutures and punctured with a 20-gauge needle. This procedure released 1–2 mm³ of feces into the peritoneal cavity. The cecum was restored to its normal position, and the abdominal incision was closed in two layers using 4-0 silk sutures. Sham-operated animals underwent the same manipulations, except for the cecal ligation and puncture. To examine the effect of bilirubin on sepsis, mice were injected with 40 mg/kg of bilirubin via the tail vein immediately after surgery. For the LPS-induced sepsis model, inflammatory sepsis was induced by intraperitoneal injection of mice with 40 mg/kg of LPS. To evaluate the effect of bilirubin on this model, mice were intravenously injected with 40 mg/kg of bilirubin immediately before LPS treatment. Bilirubin was solubilized in 0.2 N NaOH, then diluted and neutralized in PBS. Control animals were injected with an equivalent volume of PBS. Mice were monitored for survival every 12 h over a total period of 96 h. The study included groups treated with antibiotics (intraperitoneal injection of 50 mg/kg ceftriaxone plus 35 mg/kg metronidazole) and those receiving PBS instead of antibiotics. Survival data were recorded, and Mantel–Cox survival curves were generated to compare survival rates across different treatment groups. At the experimental endpoint, all mice were euthanized by CO₂ inhalation following institutional and national ethical guidelines.

### 2.6. Biochemical analyses in mouse serum

While mice were euthanized, blood samples were collected via cardiac puncture using heparin-coated syringes. Mice that died after sepsis induction but before the designated collection time could not undergo blood sampling and were therefore excluded from the biomarker analysis. After the samples were centrifuged at 2000 × *g* for 30 min at 4 °C, the supernatants were collected as sera and stored at −80 °C until analysis. Biochemical analyses were performed using the Human IFN-γ Quantikine ELISA Kit (R&D Systems; Minneapolis, MN), the Human TNF-α Quantikine ELISA Kit (R&D Systems; Minneapolis, MN), the Citrullinated Histone H3 ELISA Kit (Cayman Chemical; Ann Arbor, MI), and the Quant-iT™ PicoGreen™ dsDNA Assay Kit (Invitrogen; Waltham, MA).

### 2.7. Stimulation of NETosis and measurement of NETs

To produce NETs, isolated human neutrophils (4 × 10⁶ cells) were cultured in 10 mL of RPMI 1640 medium containing 10% FBS. To stimulate NETosis, the neutrophils were treated with 250 nM PMA for 4 h or 25 μg/mL LPS for 2 h. The DNA in NETs was stained with the cell-impermeable dye PicoGreen, which detects extracellular DNA as a marker of NETosis. Fluorescence measurements were conducted at 485 nm excitation and 528 nm emission using an Infinite M1000Pro microplate reader (Tecan; Männedorf, Switzerland). To determine the fluorescence intensity of total DNAs, cells were totally lysed with 0.5% Triton X-100 for 10 min to ensure the release of all DNAs. The degree of NETosis was evaluated by calculating the ratio of the extracellular DNA intensity to total DNA intensity. Each experiment was performed in triplicate at room temperature.

### 2.8. In situ analysis of NETs

To observe NETosis *in situ*, human neutrophils were isolated and cultured on poly-L-lysine-coated coverslips, treated with 100–250 nM PMA for 4 h to induce NETosis, and fixed with 4% paraformaldehyde for 20 min. The cells were rinsed twice with PBS and incubated with 1% bovine serum albumin (BSA) for 30 min to block non-specific antibody interactions. The samples were then incubated with anti-citrullinated histone H3 antibody (diluted 1:1000) at 4 °C for 16 h. On the following day, DNA was stained with 1.5 μM SYTOX Green for 20 min, and nuclei were stained with 4′,6-diamidino-2-phenylindole (DAPI) for 5 min. Fluorescence was preserved using FluorSave Reagent. Finally, the samples were visualized under a ZEISS LSM-800 confocal microscope at 20× magnification. The fluorescence intensity was quantified using the Zen 3.7 (Blue Edition) analysis software. The stained zones were identified at a threshold to distinguish fluorescent signals from the background, and the pixel areas of the fluorescent signal were calculated using the Zen 3.7 software. The total area of stained zones in the NETosis condition was expressed as the fold change versus the area in the unstimulated condition.

### 2.9. Immunoblotting

Isolated human neutrophils and HEK293T cells were lysed by heating them at 95 °C in SDS sample buffer for 10 min. Cell lysates were loaded onto SDS-polyacrylamide gels for electrophoresis, and proteins were transferred onto Immobilon® Transfer Membranes (EMD Millipore Corporation; Billerica, MA). The membranes were blocked in skim milk and then incubated overnight at 4 °C with one of primary antibodies against NOX2, H3cit, histone H3, FLAG tag, and GAPDH. The blots were then incubated with HRP-conjugated secondary antibodies for 1 h at room temperature. Immune-reactive protein bands were detected using the ECL-Plus kit (Amersham Biosciences; Piscataway, NJ) and visualized on X-ray film. Protein expression levels were quantified by measuring the intensity of the hybridization signals using ImageJ software (NIH; Bethesda, MD).

### 2.10. Quantitative RT-PCR

RNAs were extracted using TRIzol™ reagent (Invitrogen; Carlsbad, CA). The extracted RNAs were reverse-transcribed at 42 °C for 60 min in a reaction mixture containing M-MLV Reverse Transcriptase (Enzynomics; Daejeon, Korea), an RNase inhibitor, dNTPs, and random primers. Real-time PCR was performed using the qPCR Master mix kit (Enzynomics; Daejeon, Korea), and the fluorescence emitted by the dye-DNA complex was monitored with a CFX Connect Real-Time Cycler (BIO-RAD; Hercules, CA). The mRNA levels of target genes were normalized to the GAPDH mRNA level for each sample. All reactions were performed in triplicate to ensure reliability and reproducibility of the results. The sequences of primers are listed in Supplementary Table 1.

### 2.11. H_2_O_2_ measurement in neutrophils

The cellular levels of H₂O₂ were measured using the Amplex Red Hydrogen Peroxide/Peroxidase Assay Kit (Invitrogen; Carlsbad, CA). The Amplex Red reagent (10-acetyl-3,7-dihydroxyphenoxazine), in the presence of HRP, reacts with H₂O₂ in a 1:1 stoichiometry to produce the red-fluorescent oxidation product resorufin. The level of resorufin was analyzed by measuring the intensity of emitted fluorescence. Human neutrophils were incubated with the reaction mixture containing Amplex Red reagent and HRP at 37 °C for 30 min in the dark. The fluorescence intensity was measured using a TECAN Infinite M200 Pro (Grödig, Austria). Fluorescence excitation was set at 530 nm, and emission was detected at 590 nm to quantify H₂O₂.

### 2.12. Measurement of NOX2 activity in human neutrophils

Since NOX2 catalyzes the conversion of NADPH to NADP⁺, the consumption of NADPH reflects the enzymatic activity of NOX2. Isolated human neutrophils (6 × 10⁴ cells per well) were seeded into a 96-well plate. The assay was performed in a reaction buffer (100 µL) containing 0.05 M potassium phosphate buffer (pH 7.4), PBS, and 1 mM NADPH. The consumption of NADPH was monitored by measuring the decrease in absorbance at 340 nm using a UV/VIS spectrophotometer or a microplate reader. Reactions were initiated by the addition of NADPH, and the decrease in absorbance was recorded at 30-min intervals for 3 h at 37 °C. The rate of NADPH consumption was calculated by determining the slope of the linear portion of the absorbance decay curve. All assays were performed in triplicate to ensure accuracy and reproducibility. Data were analyzed and expressed as the rate of NADPH consumption.

### 2.13. Measurement of superoxide generation in human neutrophils

Superoxide generation was analyzed based on the SOD-inhibitable reduction of cytochrome c. Human neutrophils in 96-well plates (6 × 10⁴ per well) were incubated at 37 °C with 100  µM cytochrome c in HBSS buffer (pH 7.4). Cytochrome c reduction by superoxide was monitored at 550 nm in a microplate reader. Parallel reactions containing SOD (300 U/mL) were performed to calculate the superoxide-specific portion of cytochrome c reduction. Data are presented as the differences of 550 nm absorbances from those in the presence of SOD. All measurements were conducted in triplicate to ensure accuracy and reproducibility.

### 2.14. Surface plasmon resonance (SPR)

Surface plasmon resonance (SPR) experiments were conducted using the Biacore T200 instrument equipped with a CM5 sensor chip (GE Healthcare; Chicago, IL). NOX2 101–205 fragment protein was used as the ligand, and bilirubin as the analyte. The running buffer consisted of 10 mM HEPES (pH 7.4), 150 mM NaCl, 3 mM EDTA, 0.005% (v/v) Tween-20, and bilirubin (0.125–1 µM). The sensor chip was activated using a 1:1 mixture of 0.2 M N-Ethyl-N-dimethylaminopropyl carbodiimide hydrochloride (EDC) and 0.05 M N-hydroxy succinimide for 7 min at a flow rate of 10 µL/min. NOX2_101–205 fragment was immobilized on the activated chip at a concentration of 100 ng/mL in 10 mM sodium acetate (pH 4.0) with a flow rate of 5 µL/min until a response of approximately 700 RU was reached. Following immobilization, any remaining reactive groups were blocked by injecting 1 M ethanolamine-HCl (pH 8.5) at a flow rate of 10 µL/min for 7 min. Bilirubin solutions were injected over the NOX2_101–205-immobilized sensor surface at a flow rate of 30 µL/min for 2 min, followed by a dissociation phase of 2 min. Regeneration of the sensor surface between injections was achieved using 10 mM NaOH at a flow rate of 30 µL/min for 30 s. Data were collected at a sampling rate of 1 Hz, and the resulting sensor grams were analyzed using the Biacore T200 Control Software. Kinetic parameters were determined using a 1:1 Langmuir binding model to calculate the association rate constant (*K_a_*), the dissociation rate constant (*K_x_*), and the equilibrium dissociation constant (*K_d_*). Negative controls involving the injection of running buffer without analyte were performed to establish baseline response levels and confirm the absence of non-specific binding. Each analyte concentration was tested in triplicate to ensure the reproducibility and reliability of the experimental results.

### 2.15. Molecular docking and interaction analysis

The interaction between NOX2 and bilirubin was analyzed using the protein-ligand docking tool CB-DOCK2 (https://cadd.labshare.cn/cb-dock2/php/index.php) [38]. This tool leverages AutoDock Vina [39,40], a widely used molecular docking algorithm that predicts the optimal binding pose of ligands to their target proteins by evaluating binding affinities through an efficient scoring function. The three-dimensional structure of the full-length human NOX2 protein was obtained from the AlphaFold Protein Structure Database (https://alphafold.ebi.ac.uk) [41], and the structure of bilirubin was retrieved from the PubChem database (PubChem CID: 5280352). NOX2 and bilirubin were imported into CB-DOCK2, where docking simulations were conducted under default configurations provided by the tool, ensuring unbiased exploration of potential binding sites. Subsequently, interaction analysis was carried out using the 2D pose view feature of the ProteinsPlus platform (https://proteins.plus) [42]. The 3D visualization of the binding site was performed using UCSF Chimera, which facilitated highlighting the structural characteristics of the model and preparing presentation materials [43]. ProteinsPlus was further utilized to conduct a detailed analysis of the interactions between bilirubin and NOX2.

### 2.16. Statistical analysis

The survival rates were determined using the Mantel–Cox method, and significant differences in mortality were assessed with the log-rank test. Data were expressed as the means ± SD (or SEM). Statistical significance was analyzed using the unpaired Student's *t*-test or Mann–Whitney U test in the GraphPad Prism 5.3 software, and *p* < 0.05 was considered statistically significant in a two-tail test. Each experiment was performed at least three times independently to ensure reliability.

## 3. Result

### 3.1. Bilirubin improves mouse survival by inhibiting NETosis in CLP- or LPS-induced sepsis model

**Figure 1.**
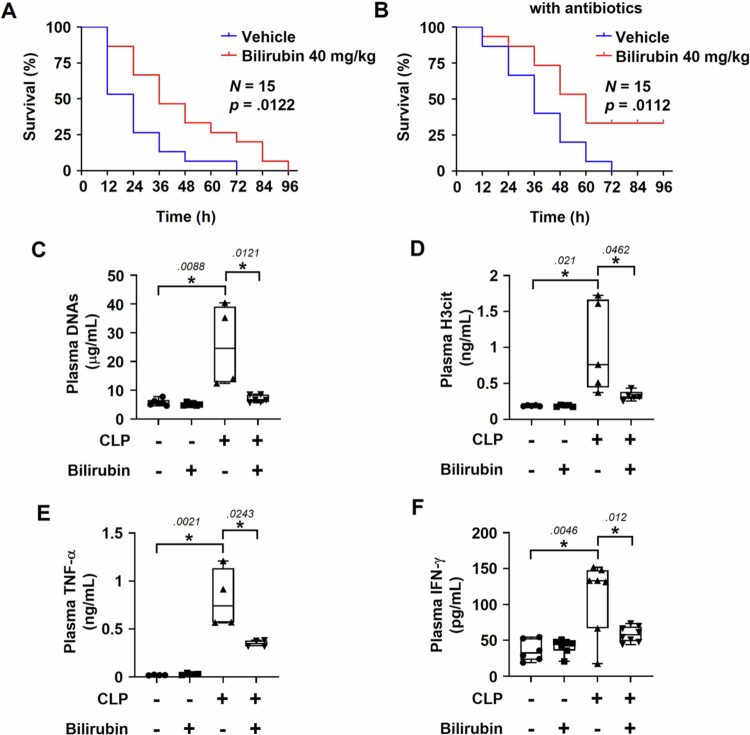
Effects of bilirubin against CLP-induced sepsis in mice. (A) Mice were subjected to CLP surgery and intravenously injected with bilirubin (40 mg/kg) or vehicle. Mice were monitored at 12-h intervals to check the survival rates. (B) After CLP surgery, all mice received intraperitoneal injection of an antibiotics mixture (ceftriaxone 50 mg/kg and metronidazole 35 mg/kg). Mice were then intravenously administered either bilirubin (40 mg/kg) or vehicle. The state of survival was monitored for 96 h. The survival rates between the bilirubin and the vehicle groups were statistically analyzed using the Mantel-Cox test (*N* = 15). (C, D) Biomarkers for NETosis were measured in the plasma of sham or CLP mice treated with vehicle or bilirubin 12 h after injection. Cell-free DNAs were stained with PicoGreen (C) and the H3cit levels were assessed using the ELISA kit (D). (E, F) Pro-inflammatory cytokines TNF-α (E) and IFN-γ (F) were analyzed in the plasma of the mice using specific ELISA kits. Each bar represents the mean ± SEM (*N* = 5–8). Asterisks and numbers represent statistical significance and *p-*values by Mann–Whitney U test, respectively.

The cecal ligation and puncture (CLP) procedure was employed to mimic sepsis in mice [44]. Bilirubin (40 mg/kg) was once injected immediately after the CLP procedure. The plasma levels of bilirubin in the control and the bilirubin-treated groups were 0.827 mg/dL and 2.438 mg/dL, respectively (Supplementary Figure S1). Mantel–Cox survival analysis revealed that bilirubin significantly increased survival rates compared to the control group, strongly indicating that bilirubin plays a protective role in sepsis (Figure 1A). When the dose of bilirubin was reduced by half (20 mg/kg), the survival rate was also shown to increase. However, there was no statistically significant difference between the bilirubin and vehicle groups. (Supplementary Figure S2A). To rule out the possibility that bilirubin reduces bacterial burden in the peritoneum, the growth of bacteria was controlled by intraperitoneally injecting antibiotics (50 mg/kg ceftriaxone plus 35 mg/kg metronidazole) after the CLP procedure. Nonetheless, bilirubin effectively improved mouse survival under sepsis (Figure 1B). The effect of bilirubin against sepsis may be irrelevant to bacterial growth.

How does bilirubin improve mice survive under sepsis? We hypothesized that bilirubin suppresses inflammation-related lethal processes by inhibiting NETosis. The plasma levels of extracellular DNAs and citrullinated histone H3 (H3cit), both of which are key components of NETs, were noticeably enhanced in CLP-subjected mice, but both the levels were significantly reduced by bilirubin (Figure 1C and D). These results suggest that bilirubin inhibits excessive NETosis in septic conditions. In addition, the plasma levels of pro-inflammatory cytokines TNF-α and IFN-γ rose in the CLP-induced sepsis model, which was attenuated by bilirubin (Figure 1E and F). Collectively, bilirubin seems to mitigate sepsis by inhibiting NETosis and inflammatory reaction.

**Figure 2.**
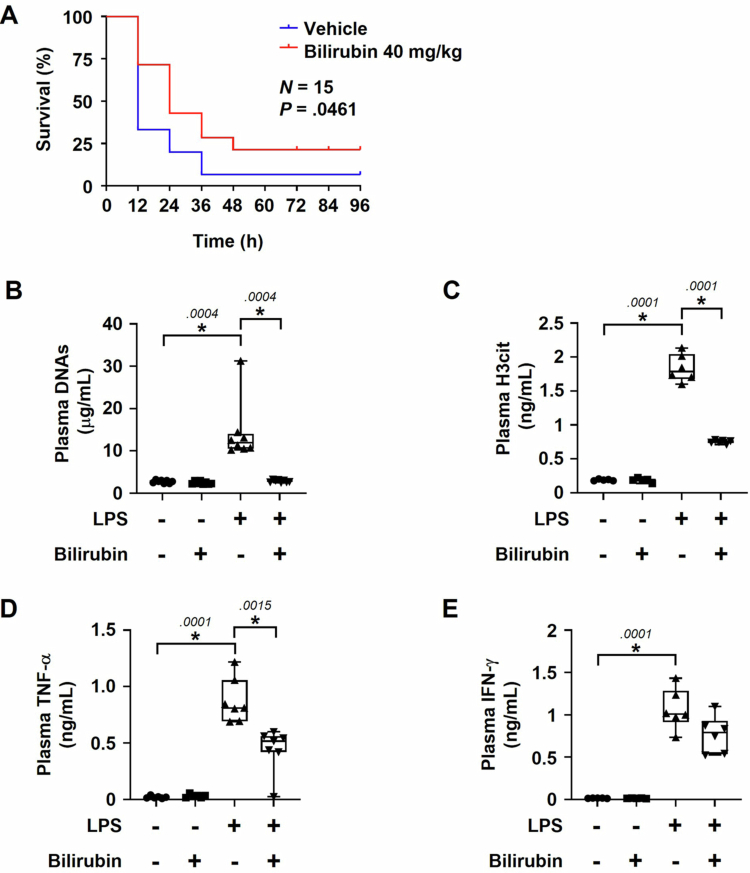
Effects of bilirubin against LPS-induced sepsis in mice. (A) Mice were intraperitoneally injected with 40 mg/kg of LPS and intravenously injected with bilirubin (40 mg/kg) or vehicle. Mice were monitored at 12-h interval to check the survival rates. The survival rates were statistically analyzed using the Mantel-Cox test (*N* = 15). (B, C) NETosis markers were measured in the plasma of PBS control or LPS-treated mice 12 h after bilirubin or vehicle injection. The plasma levels of cell-free DNAs and H3cit were analyzed using PicoGreen (B) and the ELISA kit (C), respectively. (D, E) Pro-inflammatory cytokines TNF-α (D) and IFN-γ (E) were analyzed in the plasma using specific ELISA kits. Each bar represents the mean ± SEM (*N* = 5–8). Asterisks and numbers represent statistical significance and *p-*values by Mann–Whitney U test, respectively.

In addition to CLP, LPS is also widely used to mimic sepsis in mice [44]. Mice were intraperitoneally injected with LPS (40 mg/kg) and also intravenously injected with bilirubin (40 mg/kg) or vehicle. Consequently, while one of fifteen mice in the control group survived 36 h after LPS treatment, four of fifteen mice in the bilirubin group could survive under the same conditions. Mantel–Cox survival analysis revealed that bilirubin significantly increased survival rates after LPS treatment (Figure 2A). We also checked whether bilirubin negatively regulates NETosis and inflammatory reaction in the LPS-induced sepsis model. As expected, the plasma levels of extracellular DNAs, H3cit, TNF-α, and IFN-γ were substantially enhanced in LPS-treated mice. All the markers in LPS-treated mice were significantly reduced by bilirubin (Figure 2B–E). However, bilirubin at a lower dose (20 mg/kg) did not significantly improve the survival rate in the LPS model (Supplementary Figure S2B). These results suggest that bilirubin inhibits NETosis and the production of pro-inflammatory cytokines in mice treated with LPS. Therefore, bilirubin is likely to ameliorate the LPS-induced shock by inhibiting NETosis and inflammatory reaction.

### 3.2. Bilirubin inhibits NETosis in activated neutrophils

NETosis in neutrophils can be induced by exogenous chemicals such as phorbol 12-myristate 13-acetate (PMA) and lipopolysaccharide (LPS) [45]. PMA activates NOX2 by stimulating protein kinase C, leading to ROS generation [46]. Then, ROS activate peptidylarginine deiminase 4 (PAD4), which catalyzes the histone citrullination and subsequently promotes chromatin decondensation to induce NETosis [23]. LPS activates neutrophils through Toll-like receptor 4 [47]. LPS also induces NETosis by triggering intracellular signaling pathways, which are also reinforced by NOX2-driven ROS [46].

**Figure 3.**
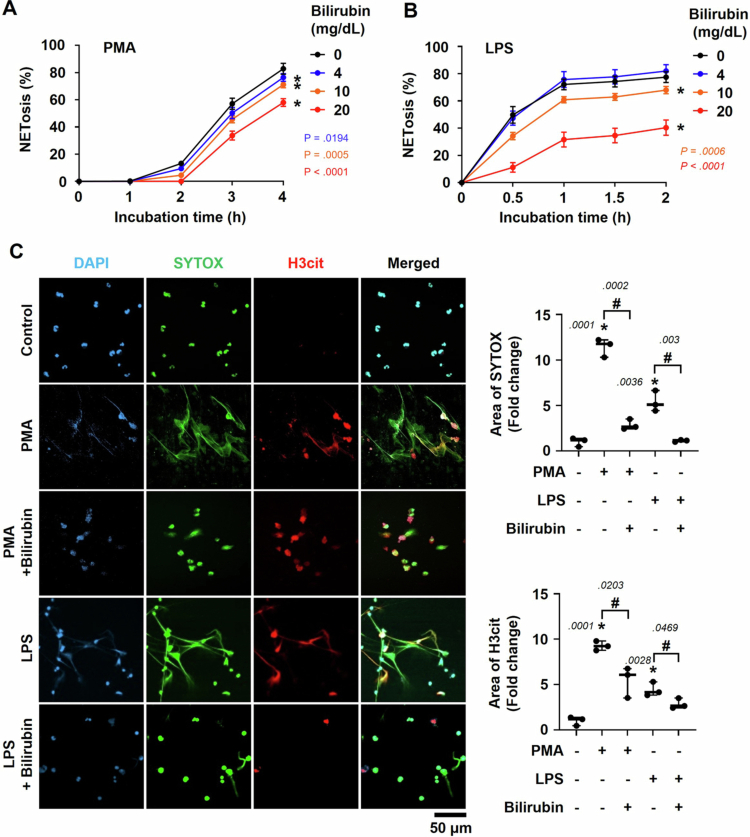
Inhibitory effect of bilirubin on NETosis in human neutrophils. (A, B) NETosis in human neutrophils was induced using two stimulants PMA (250 nM) and LPS (25 μg/mL) in the presence of bilirubin (4, 10, or 20 mg/dL). NETs were stained with PicoGreen and the degree of NETosis was quantified as the ratio of extracellular DNA level to total DNA level. Each symbol represents the mean ± SD (*N* = 5). * denotes *p* < 0.05 versus the control group by Student's *t*-test. (C) Neutrophils were treated with PMA or LPS in the presence or absence of bilirubin. They were stained with DAPI (nuclei, blue) and SYTOX Green (extracellular DNA, green), and subjected to immunofluorescence (red) with anti-H3cit antibody. Representative confocal microscopy images are presented in the left panel. The degree of NETosis is evaluated by analyzing the area of structures positively stained with SYTOX Green or immunostained with anti-H3cit antibody. The stained areas were assessed using the Zen 3.7 (Blue Edition) program and plotted as bar graphs (the mean ± SD, *N* = 3) in the right panel. * and # denote statistical significances (Student's *t*-test) versus the untreated group and the PMA- or LPS-treated group, respectively. *p*-values are indicated adjacent to the significance marks.

To examine the effect of bilirubin on NETosis, human neutrophils were subjected to NETosis in the presence of 250 nM PMA or 25 µg/mL LPS. Consequently, bilirubin (4, 10, or 20 mg/dL) inhibited NETosis in a dose-dependent manner (Figure 3A and B). Biliverdin, which is a precursor of bilirubin in the heme degradation pathway, is structurally similar to bilirubin. Therefore, we tested the possibility that it inhibits NETosis as well. Biliverdin was also found to inhibit NETosis to a similar extent as bilirubin (Supplementary Figure S3). To further validate such an effect of bilirubin, immunofluorescence analyses were employed to visualize NETosis. After being treated with 250 nM PMA or 25 µg/mL LPS, human neutrophils were stained with DAPI, SYTOX Green, and anti-citrullinated H3 antibody. PMA- or LPS-treated neutrophils produced amorphous structures consisting of DNAs and citrullinated H3, which are representative markers for NETs. In the stimulated neutrophils, bilirubin significantly attenuated the production of both the markers (Figure 3C). Quantitative analyses of the fluorescence signals confirmed the inhibitory effect of bilirubin on NETosis. These findings further support our notion that bilirubin acts as a negative regulator of NETosis.

### 3.3. Bilirubin inhibits NETosis irrespective of the activation of PPAR-α

As PPAR-α participates in various anti-inflammatory processes, we investigated whether the bilirubin-mediated activation of PPAR-α is responsible for the NETosis inhibition. The quantitative RT-PCR analyses revealed that bilirubin as well as fenofibrate (a PPAR-α activator) significantly upregulated the PPAR-α downstream genes, such as *PDK4* (pyruvate dehydrogenase kinase 4), *FABP1* (fatty acid-binding protein 1), and *GK* (glycerol kinase) (Supplementary Figures S4A–C). These results indicate that bilirubin acts as a ligand to activate PPAR-α. However, fenofibrate failed to block PMA- or LPS-induced NETosis in neutrophils (Supplementary Figures S4D and E). Moreover, GW6471 (a PPAR-α inhibitor) could not reverse the bilirubin's action against NETosis induced by PMA or LPS. These results suggest that bilirubin's inhibition of NETosis is not attributed to PPAR-α activation.

### 3.4. Bilirubin inhibits the enzymatic reaction and superoxide generation of NOX2

**Figure 4.**
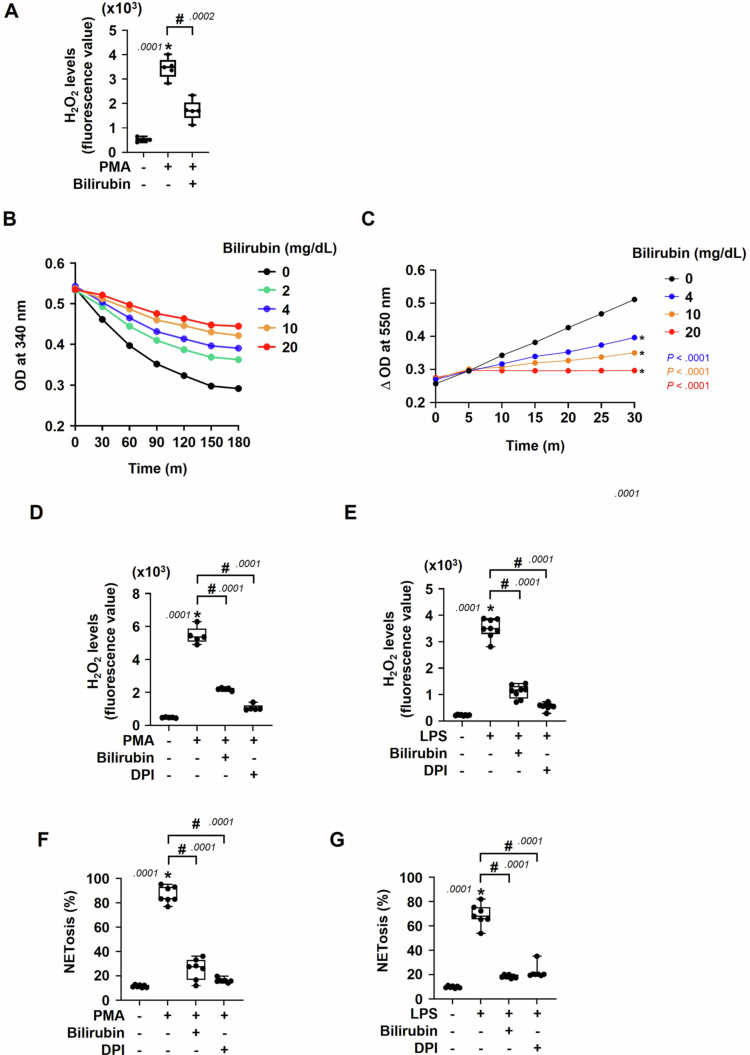
Bilirubin attenuates NETosis by inhibiting NADPH oxidase. (A) Neutrophils were treated with PMA (250 nM) for 30 min in the presence of bilirubin (20 mg/dL). H₂O₂ was detected in neutrophils using Amplex Red and HRP. (B) The NADPH oxidase activity in neutrophils was assessed based on the NADPH consumption rate. After neutrophils were incubated with 1 mM NADPH at 37 °C, the consumption of NADPH was monitored by measuring the absorbance at 340 nm. (C) The cytochrome c reduction assay was performed to evaluate superoxide production in neutrophils. Cells were incubated with 100 µM cytochrome c at 37 °C, and the reduction of cytochrome c was monitored by measuring the absorbance at 550 nm. (D, E) H₂O₂ levels were measured using Amplex Red and HRP in neutrophils treated with PMA (D) or LPS (E) in the presence of bilirubin or DPI. Data are presented as the means ± SD (*N* = 5 or more). (F, G) Neutrophils were treated with PMA (F) or LPS (G) for 30 min in the presence of bilirubin (20 mg/dL) or DPI (10 mM). NETs were stained with PicoGreen and the degree of NETosis was quantified as the ratio of NETotic DNA level to total DNA level. Statistical significance was determined using Student's *t*-test. * and # denote statistical significance versus the untreated group and the PMA- or LPS-treated group, respectively. *p*-values are indicated adjacent to the significance marks.

Given that bilirubin has an antioxidant activity, we tested the possibility that the ROS-dependent process of NETosis is attenuated by bilirubin. Consequently, the intracellular level of ROS was noticeably enhanced during PMA-induced NETosis, which supports the previous scenario emphasizing a crucial role of ROS in NETosis. Interestingly, bilirubin significantly reduced the ROS level in PMA-stimulated neutrophils (Figure 4A). Since NOX2 is the main source for ROS in neutrophils, we next checked if bilirubin inhibits the enzymatic action of NOX2, which was evaluated by monitoring the consumption rate of NADPH in the culture media of neutrophils. Consequently, the consumption rate of NADPH was decreased by bilirubin in a dose-dependent manner (Figure 4B), suggesting that bilirubin inhibits the enzymatic reaction of NOX2. To confirm such an action of bilirubin to NOX2, the superoxide production from NOX2 was analyzed using cytochrome c. The SOD-inhibitable portion of reduced cytochrome c was decreased dose-dependently by bilirubin (Figure 4C and Supplementary Figure S5), further supporting the NOX2 inhibition by bilirubin. As superoxide anion is naturally converted to H_2_O_2_, we next measured H_2_O_2_ in neutrophils treated with PMA or LPS. H_2_O_2_ production was markedly enhanced by either PMA or LPS, which was significantly attenuated by bilirubin (Figure 4D and E). A NOX inhibitor DPI was used to verify the NOX2-dependent generation of H_2_O_2_. As aforementioned, the ROS generation from NOX2 has been known to give a cue sign for NETosis. DPI was administered to neutrophils under NETosis to validate such a role of NOX2 in our experimental setting. DPI suppressed the PMA or LPS-induced NETosis a lot, as shown in the bilirubin-treated groups (Figure 4F and G). Collectively, it is suggested that bilirubin blocks the NETosis process in activated neutrophils by suppressing ROS generation from NOX2.

### 3.5. Bilirubin downregulates NOX2 at the protein level

**Figure 5.**
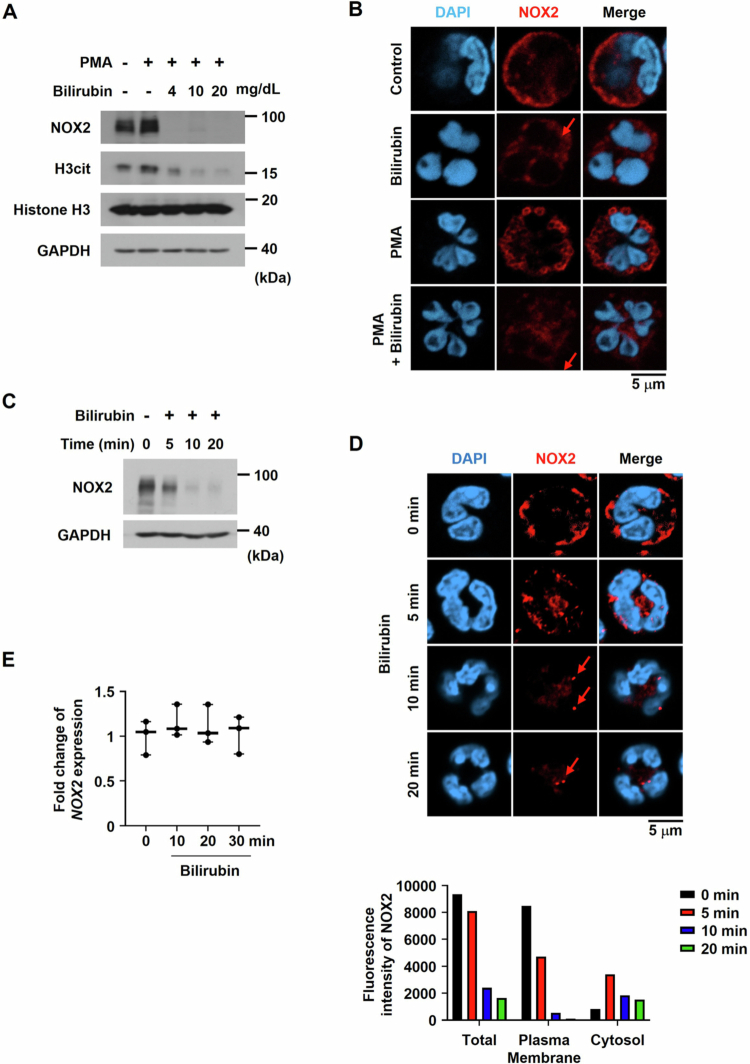
Bilirubin downregulates NOX2 at the protein level. (A) Neutrophils were incubated with PMA in the presence or absence of bilirubin at the indicated concentrations for four hours. NOX2 expression and histone H3 citrullination were analyzed by Western blotting. (B) Immunofluorescence staining of NOX2 (red) and nuclei (DAPI, blue) in neutrophils treated with 250 nM PMA and/or 4 mg/dL bilirubin. (C, D) Neutrophils were treated with bilirubin (4 mg/dL) for the indicated times. The NOX2 protein levels were analyzed by Western blotting (C) and immunofluorescence staining (D). The lower panel in (D) shows quantitative analysis of NOX2 fluorescence intensity in total, membrane, and cytosolic compartments at the indicated time points. (E) Quantitative RT-PCR analyses of NOX2 mRNAs in neutrophils treated with bilirubin (4 mg/dL) for the indicated times.

How does bilirubin inhibit the ROS production from NADPH oxidase in neutrophils? We first tested the possibility that bilirubin directly targets NOX2. Surprisingly, NOX2 protein was gone almost completely in neutrophils after 4-h treatment with bilirubin at 4 mg/dL or more concentrations (Figure 5A, Supplementary Figure S6). The inhibition of histone H3 citrullination verified the inhibitory effect of bilirubin on NETosis. To further examine such an effect of bilirubin, the expression and location of NOX2 in neutrophils were checked using the immunofluorescence analysis. Confocal images showed that NOX2 localized in the plasma membranes disappeared after bilirubin treatment in either control or PMA-stimulated neutrophils, as indicated by red arrows (Figure 5B). However, new faint signals of NOX2 were detected inside the cells of neutrophils treated with bilirubin. To check the time course of NOX2 downregulation, we treated neutrophils with bilirubin for 5–20 min and found that the NOX2 downregulation by bilirubin is almost completely done in 10 min (Figure 5C, Supplementary Figure S7). In the immunofluorescence analysis, NOX2 in the plasma membrane translocated to the cytoplasm 5 min after bilirubin treatment, appearing as puncta, as indicated by red arrows. After 10 min, the levels of NOX2 in the cytoplasm were significantly decreased (Figure 5D, upper panel). To further quantify this redistribution, NOX2 fluorescence intensity was measured in the plasma membrane and cytoplasm compartments. After bilirubin treatment, the NOX2 level was quickly reduced in the plasma membrane (Figure 5D, lower panel). In the cytoplasm, however, the NOX2 level was increased 5 min after bilirubin treatment but reduced later. However, the mRNA levels of NOX2 were not altered by bilirubin (Figure 5E), indicating that bilirubin downregulates NOX2 at the post-translational level. These results suggest that bilirubin induces the membrane-to-cytoplasm translocation of NOX2 and the degradation of NOX2 in the cytoplasm.

### 3.6. Identification of the bilirubin-dependent degradation domain within NOX2

**Figure 6.**
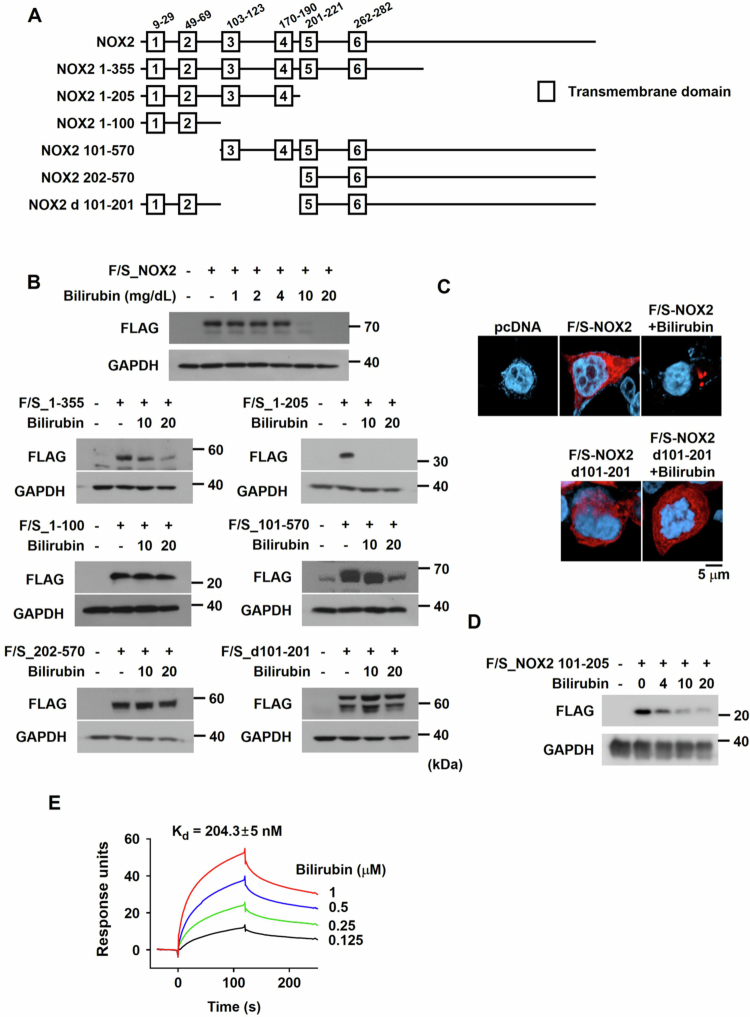
Identification of the bilirubin-dependent degradation domain in NOX2. (A) Schematic illustration of the protein structures of NOX2 and its deletion mutants. In a symbol of NOX2 a and b, ‘a’ and ‘b’ are the numbers of the first and the last amino acids in the corresponding protein. In NOX2 d101–201, the segment of amino acids 101 to 201 was deleted in the wild type NOX2. The transmembrane domains of NOX2 are marked with square boxes. (B) HEK293T cells were transfected with one of the various NOX2 plasmids. After 48 h, cells were treated with bilirubin (10 or 20 mg/dL) and subjected to Western blotting with anti-FLAG antibody. (C) Immunofluorescence staining of the NOX2 WT and the mutant lacking aa. 101–201 ectopically expressed in HEK293T cells treated with or without bilirubin (10 mg/dL). (D) HEK293T cells were transfected with 101–205 NOX2 fragment plasmids. After 48 h, cells were treated with bilirubin (4, 10 or 20 mg/dL) and subjected to Western blotting with anti-FLAG antibody. (E) SPR analysis of bilirubin binding to the His-tagged NOX2 101–205 fragment purified from *E. coli*. Bilirubin solutions at the indicated concentrations were injected over the NOX2 fragment-immobilized sensor surface for 2 min, followed by a dissociation phase of 2 min. The equilibrium dissociation constant *K_d_* was analyzed using the Biacore T200 Control Software.

Given the acute response of NOX2 to bilirubin, we examined whether bilirubin directly targets NOX2. To search for a specific domain responsible for the bilirubin's effect, a variety of NOX2 mutants were constructed as shown in Figure 6A. Considering that NOX2 is a membrane-anchored protein, the mutation was designed based on the transmembrane domains. Practically, primary neutrophils are not suitable for gene expression because they are hardly transfected with pcDNA-based vectors. Thus, we checked NOX2 and its mutated proteins in HEK293T cells with high transfection efficiency instead of neutrophils (Supplementary Figure S8). FLAG/SBP-tagged wild-type NOX2 was expressed in HEK293T (Supplementary Figure S9), and it was also effectively downregulated by bilirubin (Figure 6B, the 1st row). NOX2_1-355 and NOX2_1-205 were still downregulated by bilirubin (Figure 6B, the 2nd row), but a shorter construct NOX2_1-100 showed no response to bilirubin (Figure 6B, the 3rd row). Next, we designed the NOX2 deletion toward the C-terminal site and expressed the mutants. Consequently, NOX2_101–570 was downregulated by bilirubin, but NOX2_202-570 was not (Figure 6B, Supplementary Figure S9). These results indicate that the segment ranging from aa. 101 to aa. 201 is a potential domain responsible for the bilirubin-dependent NOX2 degradation. To confirm the bilirubin-response domain, we deleted the very site from the full-length construct of NOX2. The NOX2 lacking the site (designated NOX2_d101–201) was not downregulated at all by bilirubin (Figure 6B, the 4th row). In immunofluorescence analysis, NOX2_d101–201 was neither translocated nor degraded by bilirubin (Figure 6C, Supplementary Figure S10), whereas the wild-type NOX2 showed a sensitive response to bilirubin. Since the 101–201 segment forms an extracellular long loop (known as loop C), we hypothesized that the segment is directly targeted by extracellular bilirubin. To further evaluate the involvement of this region in NOX2 degradation, we expressed the NOX2_101–205 fragment in HEK293T cells. The cellular level of this fragment was marked reduced by bilirubin (Figure 6D), strongly indicating that the aa. 101–205 segment is essential for bilirubin-dependent NOX2 degradation. To recheck this possibility, we performed SPR analysis with a recombinant NOX2_101–205 protein (Supplementary Figure S11) and bilirubin, which revealed that bilirubin physically binds to this NOX2 fragment (Figure 6E). The equilibrium dissociation constant (*K_d_*), which is calculated by the association rate constant (*K_a_*) and the dissociation rate constant (*K_x_*), was as low as 204 nM, indicating a robust interaction between bilirubin and the fragment. Generally, a low *K_d_* represents a high affinity because the two parameters are in reciprocal relationship. Collectively, these findings suggest that bilirubin deregulates NOX2 by targeting a specific domain.

### 3.7. Molecular structure analysis for the interaction between NOX2 and bilirubin

**Figure 7.**
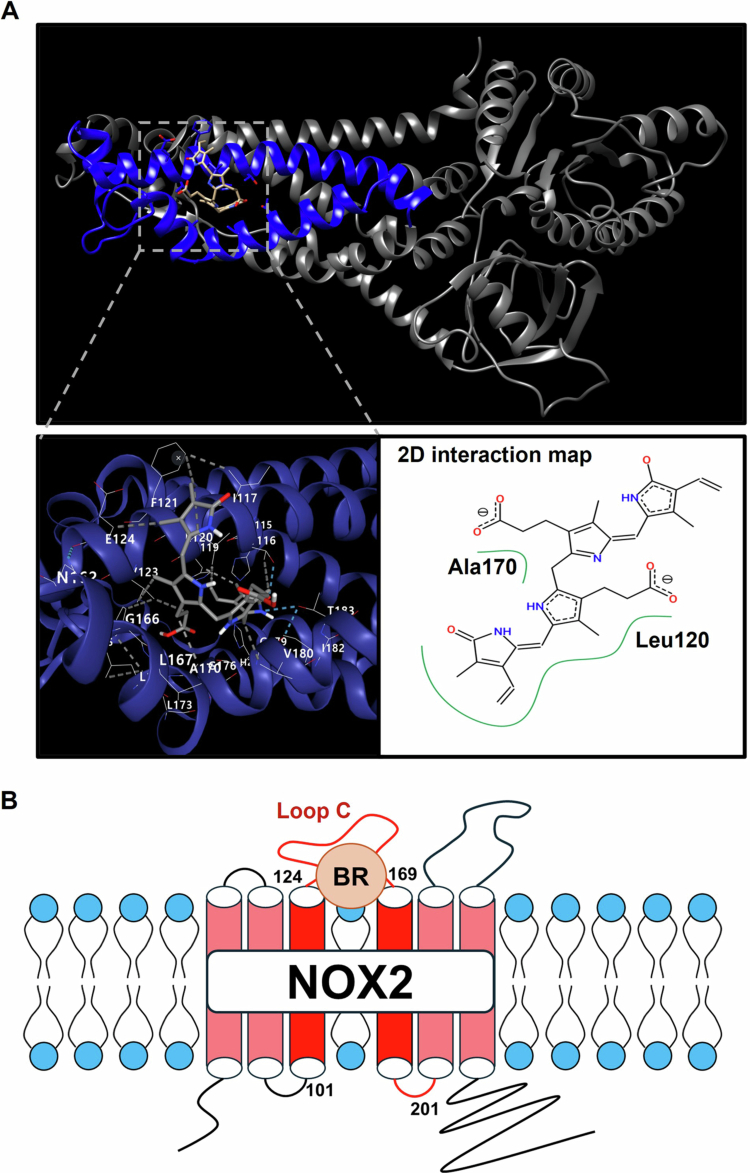
Structural modeling of NOX2 and bilirubin interaction. (A) The interaction between NOX2 and bilirubin was analyzed using CB-DOCK and visualized with UCSF Chimera and ProteinsPlus. The top panel shows a 3D structural model of NOX2 with bilirubin binding, generated using UCSF Chimera. The bottom-left panel illustrates the binding site of bilirubin on NOX2, identified through CB-DOCK analysis. The bottom-right panel displays a detailed molecular interaction diagram of bilirubin binding to NOX2, highlighting key region (e.g. Leu120–Ala170). The green-highlighted regions represent hydrophobic interactions between bilirubin and NOX2, visualized using the ProteinsPlus web-based molecular modeling tool. (B) Schematic diagram illustrating bilirubin (BR) binding to NOX2 anchored to the plasma membrane.

Bilirubin is a highly lipophilic molecule. This chemical property enables bilirubin to stably interact with proteins that possess hydrophobic binding pockets. NOX2 contains a hydrophobic pocket that can align well with the lipophilic structure of bilirubin, suggesting a high likelihood of binding to bilirubin. To test this possibility, docking studies were conducted using CB-DOCK2. In Figure 6, the aa. 101–201 domain of NOX2 is expected to bind with bilirubin. Docking simulations confirmed such an interaction because an AutoDock Vina score is as low as −7.6. The docking analysis revealed that the interaction between NOX2 and bilirubin occurs within the binding pocket spanning residues Ala109 to Ile227 (Figure 7A). The 2D interaction mapping highlights that hydrophobic interactions between the Leu120-Ala170 motif (indicated in green) and bilirubin play a crucial role in stabilizing the binding. As the Leu120-Ala170 motif is included in the loop C (Figure 7B), this structure analysis further supports our notion that the loop C is the bilirubin-dependent degradation domain of NOX2.

### 3.8. Endocytosis and autophagy are involved in NOX2 downregulation by bilirubin

**Figure 8.**
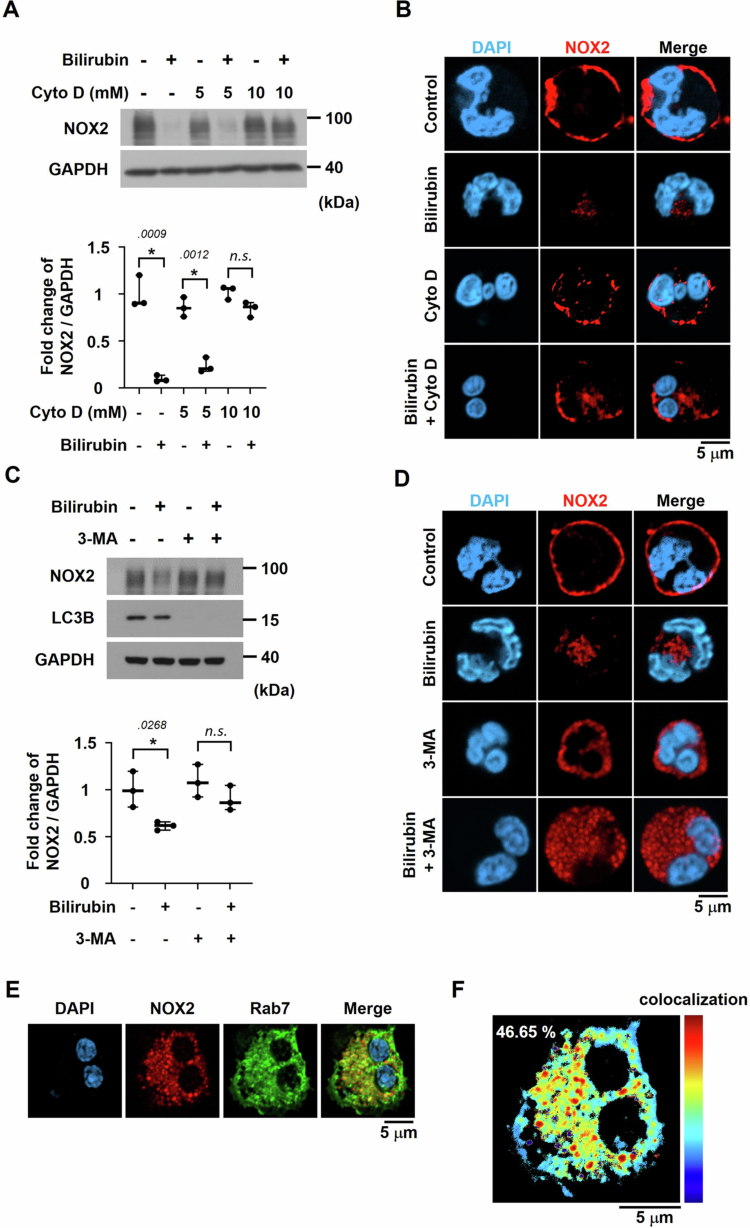
Effects of autophagy and endocytosis inhibition on bilirubin-mediated NOX2 degradation. (A) Neutrophils were treated with cytochalasin D (Cyto D) for 30 min, followed by bilirubin treatment (4 mg/dL) for 30 min. NOX2 and GAPDH expressions were analyzed by Western blotting (Top panel) and quantified using the ImageJ. The NOX2 levels are normalized by the GAPDH levels and presented as bar graphs (the means + SD, *N* = 3) in the bottom panel. (B) Immunofluorescence staining of NOX2 (red) and nuclei (DAPI, blue) in neutrophils treated with Cyto D and bilirubin under the same conditions as (A). (C) Neutrophils were treated with 3-methyladenine (3-MA, 5 mM) for 4 h, followed by bilirubin treatment (4 mg/dL) for 30 min. NOX2, LC3B, and GAPDH expressions were analyzed by Western blotting (Top panel). Protein expressions on Western blots were quantified using the ImageJ. The NOX2 levels are normalized by the GAPDH levels in the same blots and presented as bar graphs (the means + SD, *N* = 3) in the bottom panel. (D) Immunofluorescence staining of NOX2 (red) and nuclei (DAPI, blue) in neutrophils treated with 3-MA and bilirubin under the same conditions as (C). (E) Immunofluorescence staining of NOX2 (red), Rab7 (green), and nuclei (DAPI, blue) in neutrophils treated with bilirubin. (F) Colocalization analysis of NOX2 and Rab7 signals performed using ImageJ. Statistical significance was determined using Student's *t*-test. * denotes statistical significance between the indicated groups, and *n.s.* indicates no significant difference. *p*-values are indicated adjacent to the significance asterisks.

As shown in Figure 5D, NOX2 moved from the plasma membrane to the cytoplasm shortly after bilirubin treatment, and NOX2 was degraded in the cytoplasm. Given this time course, we tested the possibility that the endocytosis of the membrane-anchored NOX2 is a preceding process for NOX2 degradation. When neutrophils were treated with endocytosis inhibitor cytochalasin D (10 mM), NOX2 was no longer destroyed by bilirubin (Figure 8A, Supplementary Figure S12). In immunofluorescence imaging of neutrophils treated with cytochalasin D, NOX2 remains in the plasma membrane even in the presence of bilirubin (Figure 8B). Therefore, it is suggested that endocytosis is essential for the NOX2 downregulation by bilirubin. On the other hand, NOX2 in the plasma membrane was internalized and it appeared as puncta in the cytoplasm (Figure 5D). Cytoplasmic puncta exhibited morphological features resembling autophagosomes, suggesting a potential involvement of the autophagy-related degradation process in the bilirubin-mediated downregulation of NOX2. To examine the involvement of autophagy, neutrophils were treated with 3-methyladenine (3-MA), an inhibitor of early-stage autophagy, which attenuated the bilirubin-induced downregulation of NOX2 (Figure 8C, Supplementary Figure S13). The cellular level of LC3B (a representative marker for autophagy) was checked to examine the potential involvement of autophagy in bilirubin-induced NOX2 degradation [48]. After bilirubin treatment, NOX2-containing vesicles remain a lot in the presence of 3-MA, whereas they were shown a little in the absence of 3-MA (Figure 8D). When the autophagy process is blocked, NOX2 entrapped within the endosome may not be degraded in neutrophils. The intracellular trafficking of NOX2 was analyzed using immunofluorescence staining with an antibody against Rab7 (an endosome marker). NOX2 was observed to colocalize with Rab7 in bilirubin-treated neutrophils (Figure 8E). ImageJ analysis showed the colocalization of NOX2 and Rab7 (Figure 8F), supporting our notion that NOX2 id degraded through the endosomal/autophagic pathway. In summary, bilirubin is likely to directly target the loop C region of NOX2, which induces endocytosis of NOX2. The NOX2-containing endosomes fuse with autophagosomes to remove NOX2.

## 4. Discussion

In the present study, we demonstrated that bilirubin improved survival in mice subjected to sepsis. In two sepsis mouse models, bilirubin increased the survival rate and concomitantly reduced the plasma levels of NETosis markers. In addition, bilirubin inhibited NETosis in neutrophils activated with PMA or LPS. To understand how bilirubin inhibits NETosis, we explored two potential pathways, the activation of PPAR-α-driven gene expression and the inhibition of ROS-mediated signaling pathways. Of the two modes of action, ROS inhibition was identified as the primary mechanism responsible for the NETosis-inhibitory action of bilirubin. Mechanistically, bilirubin directly targets the extracellular loop C (aa. 101–205) of NOX2, leading to internalization and endosomal degradation of NOX2. Given that the bilirubin-dependent degradation of NOX2 was attenuated by either cytochalasin D or 3-MA, endocytosis and autophagy might be required for NOX2 degradation. A better understanding of the NETosis-inhibitory action of bilirubin will provide a new insight into the clinical management of patients with sepsis.

Mechanistically, the present study highlights the bilirubin–NOX2 regulatory axis in neutrophils. Bilirubin promoted the internalization and degradation of NOX2, leading to reduced NOX2-dependent ROS production and subsequently suppressed NETosis. Because NOX2-derived ROS is a central driver of NETosis during inflammatory responses, the regulation of NOX2 stability represents a critical upstream mechanism controlling NET formation. These findings suggest that bilirubin may function as an endogenous modulator of neutrophil inflammatory activity by limiting NOX2-driven oxidative signaling during sepsis.

In this context, we further demonstrated that endocytosis is an essential prerequisite for NOX2 degradation via the autophagic pathway. Membrane proteins like NOX2 must first be internalized through endocytosis before undergoing autophagic degradation. In addition to NOX2, this sequential mechanism is also observed in the degradation of other membrane proteins. For instance, after the epidermal growth factor receptor (EGFR) undergoes internalization following ligand binding, it is recycled back to the plasma membrane or directed to lysosomes for its degradation [49]. In this case, the internalized membrane proteins can be recycled even within the endosome. In contrast, bilirubin-bound NOX2 was found to be almost completely degraded via the autophagy pathway without recycling. This raises the question of whether bilirubin facilitates autophagy in neutrophils. In a recent study showing that bilirubin reduces liver injury in a non-alcoholic fatty liver model, bilirubin increased the expression of autophagy markers such as LC3-II and ATG7 while reducing p62 accumulation, suggesting activated autophagy [50]. Therefore, it is possible that bilirubin escalates the degradation of NOX2 in neutrophils by stimulating the autophagy process. As autophagy is involved in various signaling pathways, it is speculated that bilirubin may regulate a variety of physiological and pathological processes.

Beyond NOX2-specific regulation, we intensively investigated the inhibitory effect of bilirubin on NOX2 in neutrophil. Consequently, bilirubin was shown to bind to the second extracellular loop of NOX2, internalize NOX2, and degrade it through the autophagosome process. However, it remains unclear whether bilirubin regulates other types of NOX. The NOX family includes NADPH oxidase members (NOX1-5) and dual oxidase members (DUOX1-2) [51]. ROS generated by the enzymes serve as essential regulators of cell proliferation, differentiation, and survival [52]. However, the excessive production of ROS can contribute to the progression of various pathological conditions, including cardiovascular diseases, cancer, and neurodegenerative disorders [53–55]. Among NOX isoforms, NOX1 is the most similar to NOX2 in the amino acid sequence (65% homology), suggesting that bilirubin may also interact with NOX1. Given that NOX1 plays a significant role in the development and progression of cardiovascular diseases, inflammatory bowel diseases, and cancer, bilirubin may have therapeutic potential in these conditions [56–60]. Indeed, previous studies have shown that bilirubin reduces the incidence of cardiovascular and inflammatory bowel diseases [61–64]. Furthermore, there have been reports indicating that cancer patients with higher bilirubin levels exhibit improved survival rates [65,66]. However, the role of bilirubin in NOX1-mediated pathogenesis requires further investigation through well-designed studies.

From a translational perspective, the clinical application of bilirubin requires careful evaluation of its safety profile. In our experimental setting, the plasma level of bilirubin in mice injected with bilirubin was 2.4 mg/dL. This level is not considered severely toxic because mild hyperbilirubinemia in adults (2 to 3 mg/dL) is generally regarded as clinically benign and often observed in conditions such as Gilbert's syndrome [67]. Moreover, bilirubin is largely albumin-bound in plasma, which reduces the effective concentration of free bilirubin responsible for toxicity [68].

Despite these potential benefits, it is noteworthy that elevated serum bilirubin levels in patients with sepsis have been reported to correlate with increased disease severity and mortality [69]. However, these clinical observations do not necessarily indicate that bilirubin itself exerts harmful effects. Rather, hyperbilirubinemia could be the result from hepatic dysfunction or impaired bilirubin clearance due to systemic inflammation. In our experimental settings, bilirubin is like to prevent sepsis-induced tissue injuries by suppressing NETosis. Taken together, it is suggested that hyperbilirubinemia in severe sepsis may ameliorate septic symptoms as a compensatory response.

In addition to its effects on NETosis, neutrophils contribute to host defense through multiple mechanisms, including phagocytosis, degranulation, reactive oxygen species production, and NETosis [70]. Although NETosis plays an important role in trapping and eliminating pathogens, excessive NETosis has also been implicated in tissue injury and inflammatory pathology during sepsis [8]. Therefore, modulation of NETosis may represent a balance between antimicrobial defense and prevention of excessive inflammation. While the present study focused on the effects of bilirubin on NOX2-mediated ROS production and NETosis, bilirubin has been reported to possess antioxidant and immunomodulatory properties that may influence other neutrophil functions. Further studies will be required to determine whether bilirubin affects additional neutrophil activities such as degranulation or exocytosis during systemic inflammation.

We here demonstrated that NETosis inhibition underlies the protective effects of bilirubin in sepsis. However, NETosis inhibition should not be the only pathway responsible for the bilirubin effect. Bilirubin has been reported to exert multiple biological activities, including antioxidant and immunomodulatory effects. It acts as a potent endogenous antioxidant by scavenging reactive oxygen species, inhibiting lipid peroxidation, and suppressing NADPH oxidase–mediated ROS production [71,72]. In addition, bilirubin exerts anti-inflammatory effects by reducing pro-inflammatory cytokine production, inhibiting inducible nitric oxide synthase, and limiting leukocyte recruitment via downregulation of adhesion molecules [73,74]. Furthermore, bilirubin modulates immune responses by suppressing T cell activation, inhibiting costimulatory signaling, and downregulating MHC class II expression [75,76]. Therefore, the survival benefit observed in our models may result from the combined effects of NETosis inhibition and other bilirubin-mediated protective mechanisms.

## 5. Conclusion

In summary, we found that bilirubin inhibits NETosis in neutrophils by targeting NOX2 and suppressing ROS generation. These findings suggest that bilirubin may represent a potential therapeutic candidate for modulating inflammatory responses in sepsis, although further studies are required to evaluate its clinical applicability.

## Supplementary Material

Supplementary material ARRIVE Author Checklist.pdf

Supplementary figures and table R1.docx Supplementary figures and table R1.docx

## Data Availability

Most of the data required to evaluate the conclusions of the study are included in the main text, figures, and supplementary materials. Raw data can be provided upon request.
