# Supplementary material for: Bilirubin reduces mortality in sepsis models by inhibiting NOX2-mediated formation of neutrophil extracellular traps
Source: Redox Rep. 2026 Apr 28;31(1):2664962. doi: 10.1080/13510002.2026.2664962 (PMC13126949; doi:10.1080/13510002.2026.2664962)
Supplement: Supplementary figures and table R1.docx [file YRER_A_2664962_SM2055.docx]

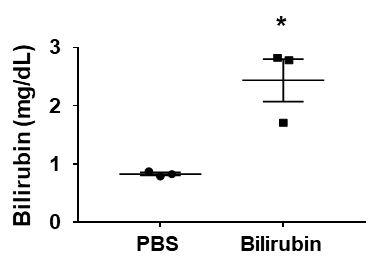


**Supplementary Figure S1. Bilirubin concentrations in mouse plasma samples**

Bilirubin levels were analyzed using the bilirubin assay kit. BALB/c mice were intravenously injected with PBS or bilirubin (40 mg/kg). Blood was collected 1 hour after injection. Data are presented as the means ± SEM (*N* = 3). Statistical significance was analyzed using Mann–Whitney U test. *: *P* < .05 versus the PBS group.


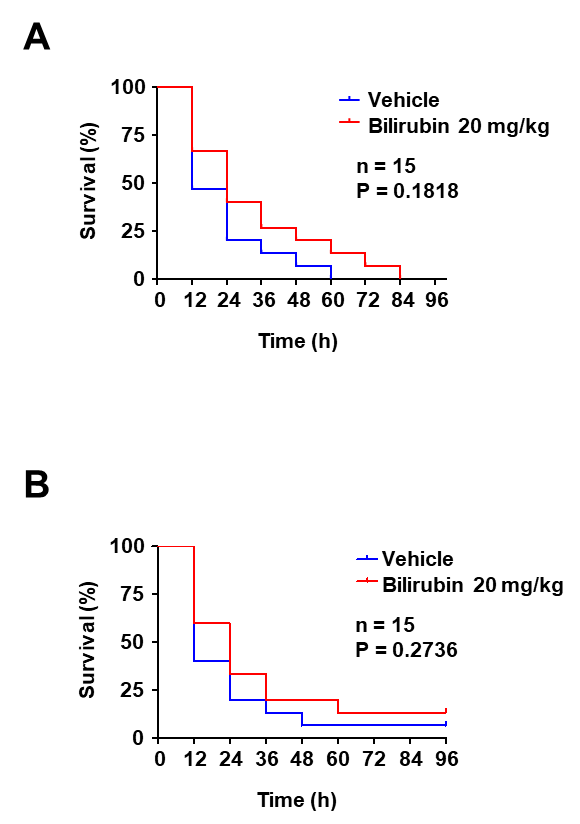


**Supplementary Figure S2. Effects of half-dose bilirubin against CLP- and LPS induced sepsis in mice**

(A) Mice were subjected to CLP surgery and intravenously injected with bilirubin (20 mg/kg) or vehicle. (B) Mice were intraperitoneally injected with 40 mg/kg of LPS and intravenously injected with bilirubin (20 mg/kg) or vehicle. Mice were monitored at 12-hour interval to check the survival rates. The survival rates were statistically analyzed using the Mantel-Cox test (N=15).


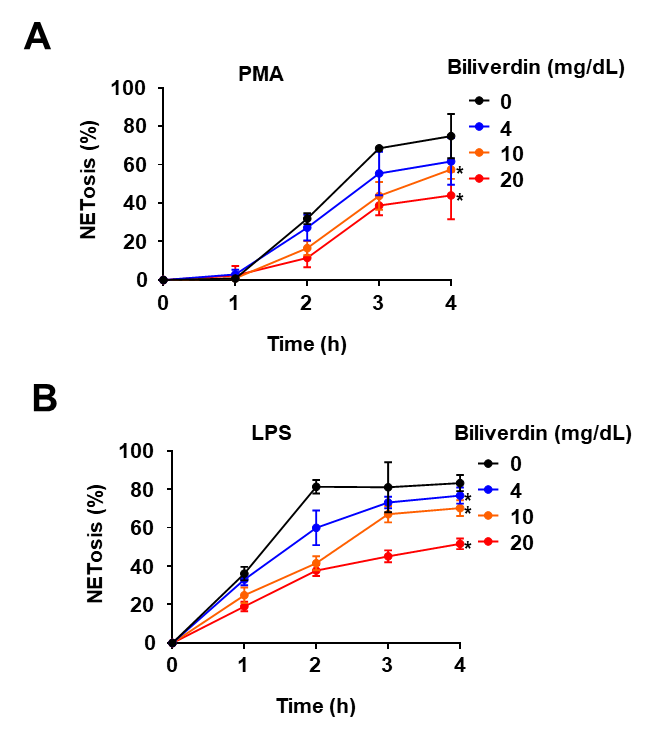


**Supplementary Figure S3. Inhibitory effect of biliverdin on NETosis in human neutrophils.**

NETosis in human neutrophils was induced using two stimulants PMA (250 nM) and LPS (25 μg/mL) in the presence of biliverdin (4, 10, or 20 mg/dL). NETs were stained with PicoGreen and the degree of NETosis was quantified as the ratio of NETotic DNA level to total DNA level. Each symbol represents the mean ± SD (N = 4). * denotes P < .05 versus the control by Student’s t-test.


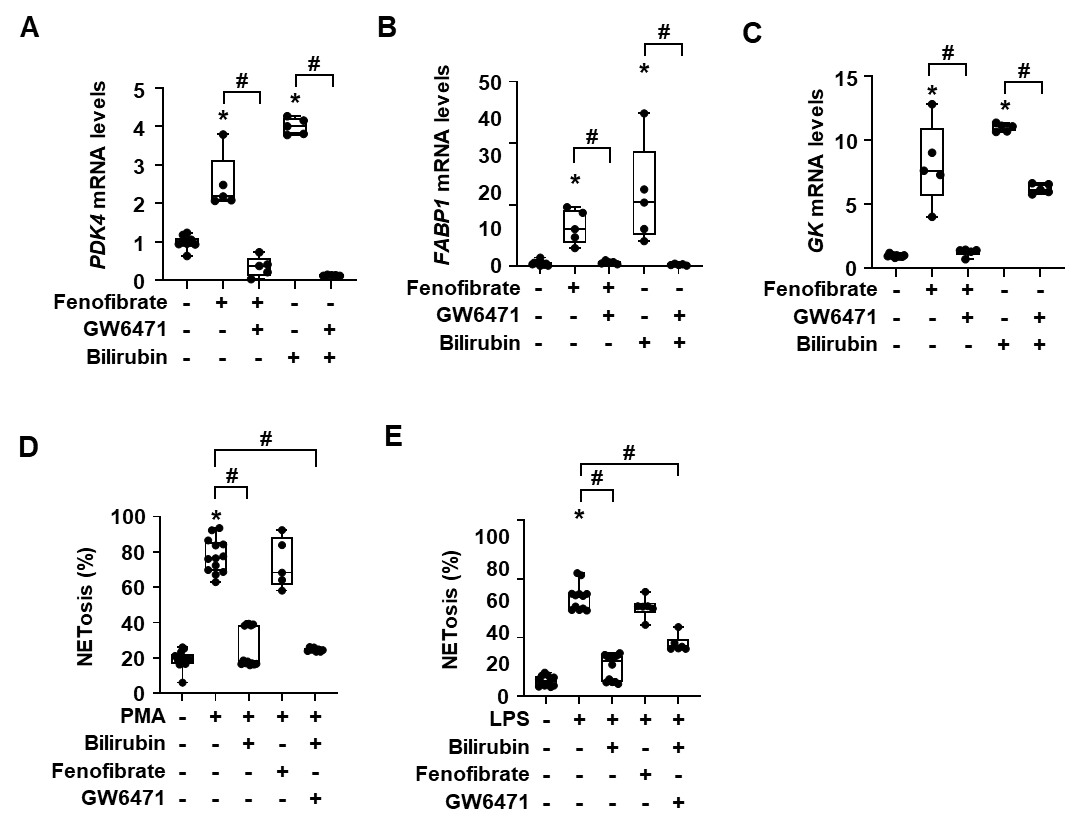


**Supplementary Figure S4. PPAR-α activation is not responsible for the bilirubin-mediated attenuation of NETosis**

(A–C) Neutrophils were pretreated with GW6471 (10 µM) for 2 hours and further treated with bilirubin (20 mg/dL) or fenofibrate (30 µM) for 4 hours. The mRNA levels of PPAR-α target genes *PDK4*, *FABP1*, and *GK* were analyzed using quantitative RT-PCR. (D, E) Neutrophils were treated with PMA or LPS in the presence or absence of bilirubin (20 mg/dL), fenofibrate (30 µM), or GW6471 (10 µM). Bilirubin and fenofibrate were administered simultaneously with PMA or LPS. NETosis was measured by PicoGreen assay. Data (the ratio of NETotic DNAs to total DNAs) are presented as the means ± SD (*N* = 5 or more). Statistical significance was analyzed using Student’s t-test. *: *P* < .05 versus the control group, #: *P* < .05 between the indicated groups.


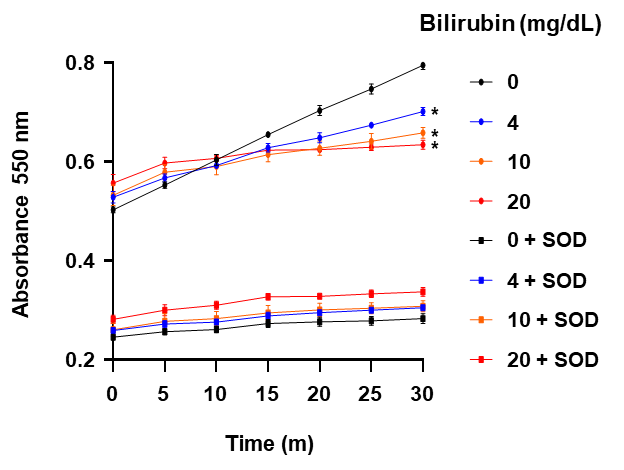


**Supplementary Figure S5. Cytochrome c reduction assay in neutrophils treated with bilirubin**

Primary neutrophils were isolated from peripheral blood and incubated with the indicated concentrations of bilirubin or vehicle control. Superoxide production was quantified by measuring the reduction of cytochrome c at 550 nm at the indicated time points. Each condition was tested in triplicate wells, and the experiment was independently repeated at least three times. The calculated Δ absorbance values from these raw data are presented in the figure 4. Data are expressed as mean ± SD (N = 3). * denotes P < .05 versus the vehicle control by Student’s t-test.


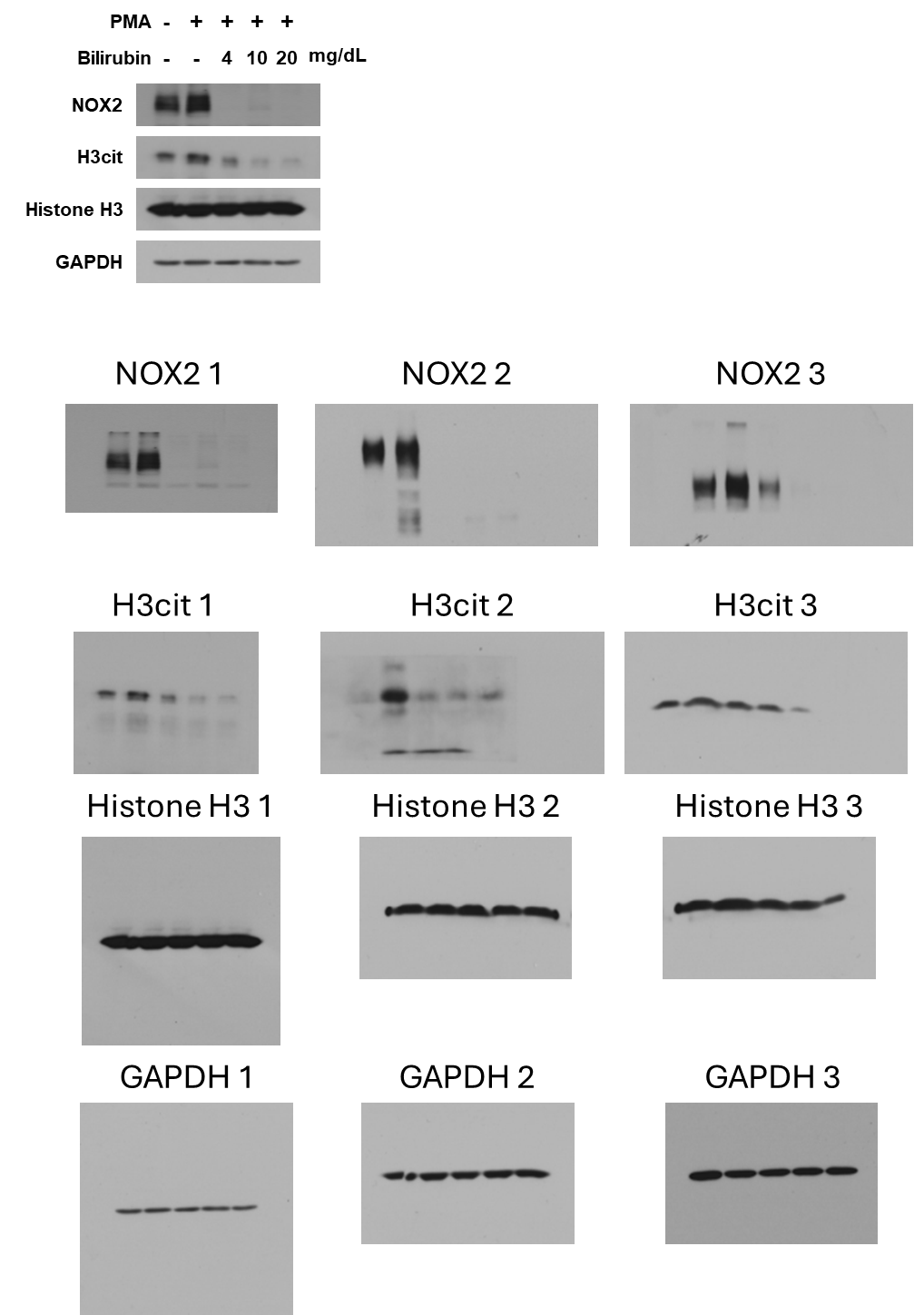


**Supplementary Figure S6. Uncropped Blots for Figure 5A**


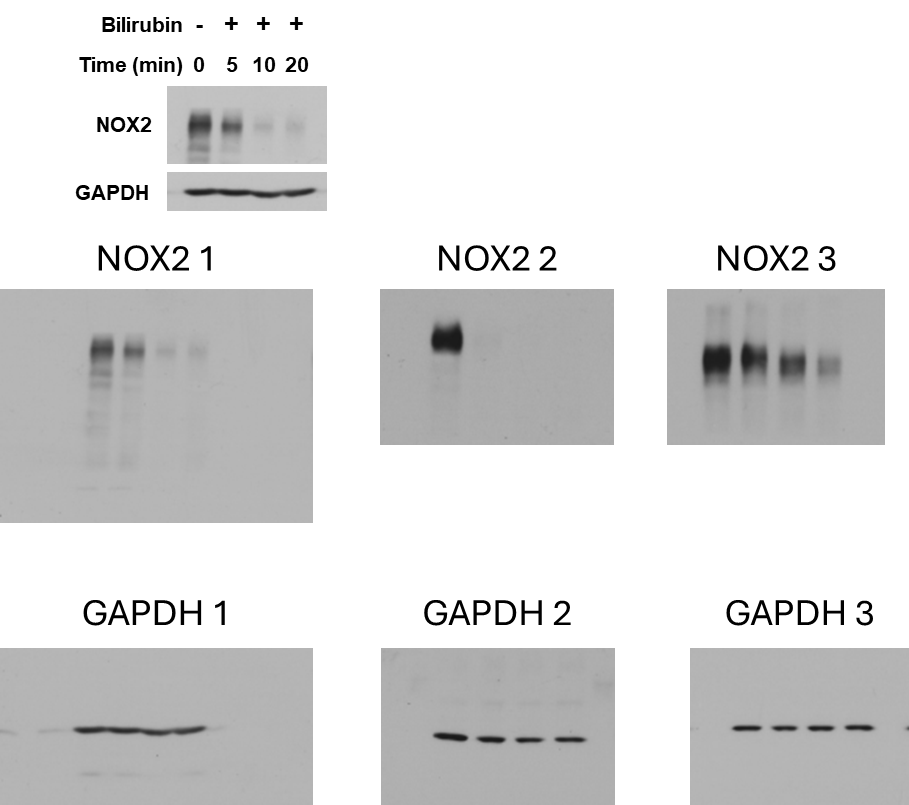


**Supplementary Figure S7. Uncropped Blots for Figure 5C**


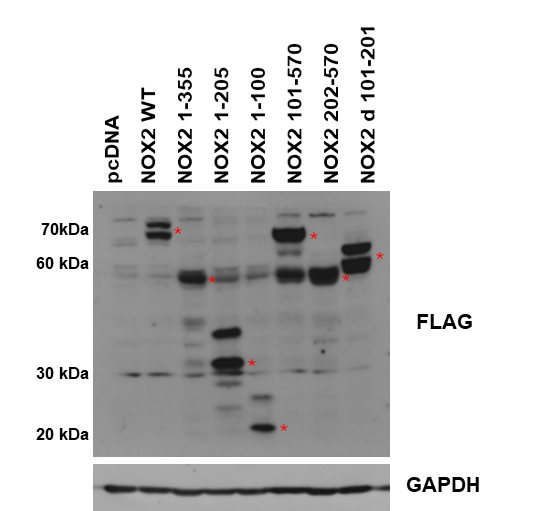


**Supplementary Figure S8. Expressions of NOX2 and its deletion mutants in 293T cells.**

Western blot analyses of wild type NOX2 and its deletion mutants (1–355, 1–205, 1–100, 101–570, 202–570, and d101–201) ectopically expressed in HEK293T cells. Flag/SBP-tagged NOX2 proteins were immunoblotted with an anti-FLAG antibody. Red asterisks indicate the specific bands of expressed proteins.


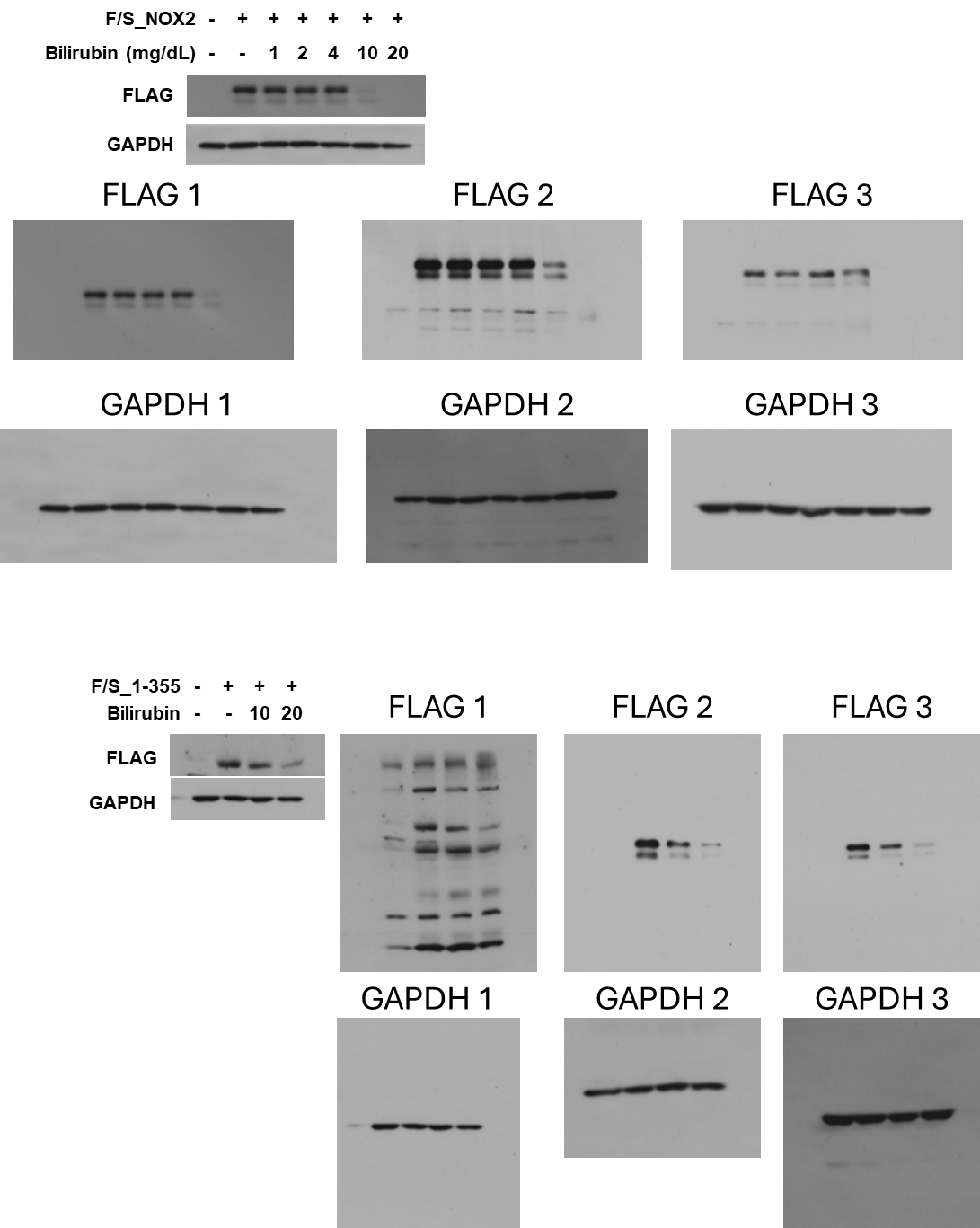


**Supplementary Figure S9. Uncropped Blots for Figure 6B**


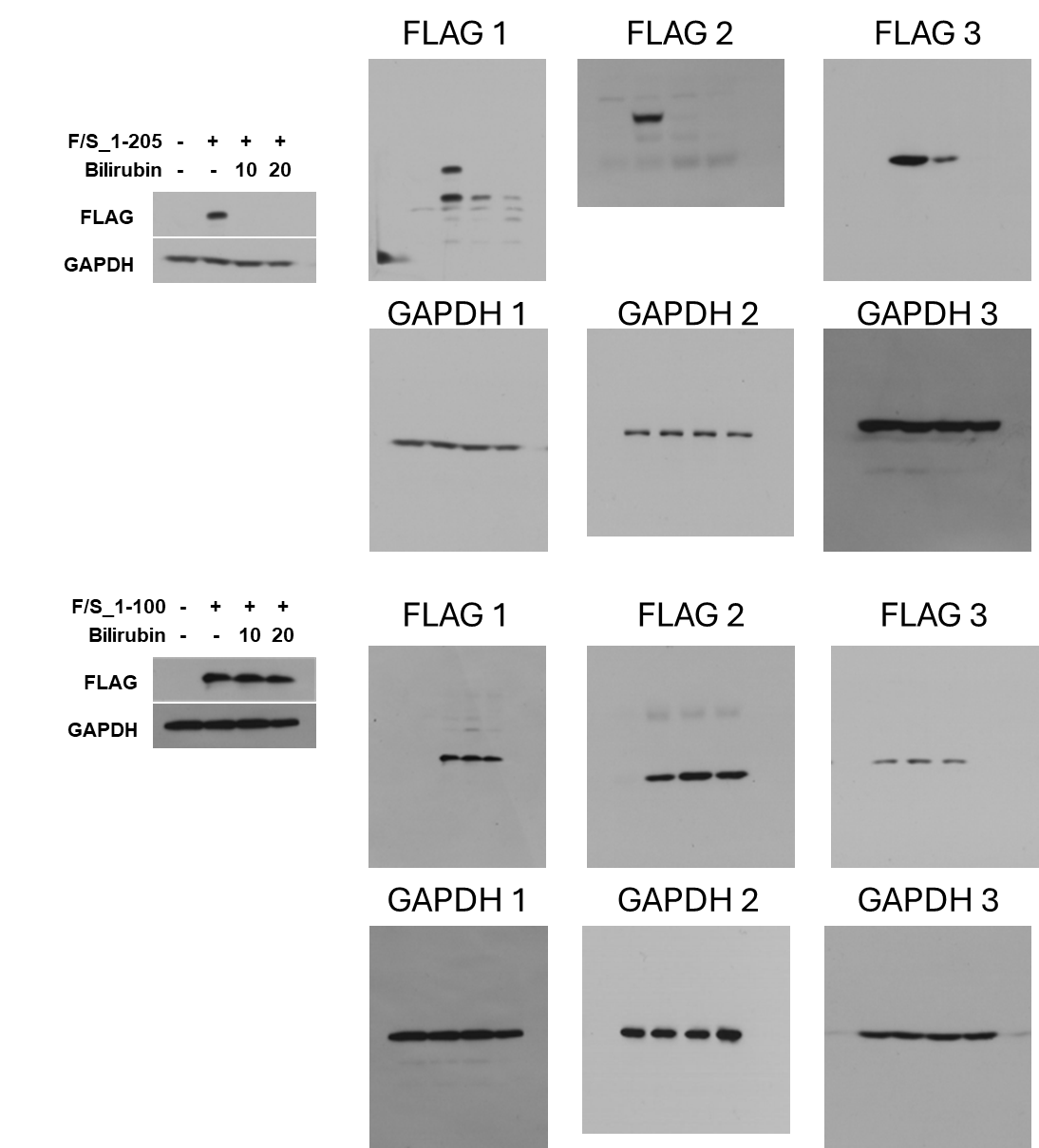


**Supplementary Figure S9. Uncropped Blots for Figure 6B**


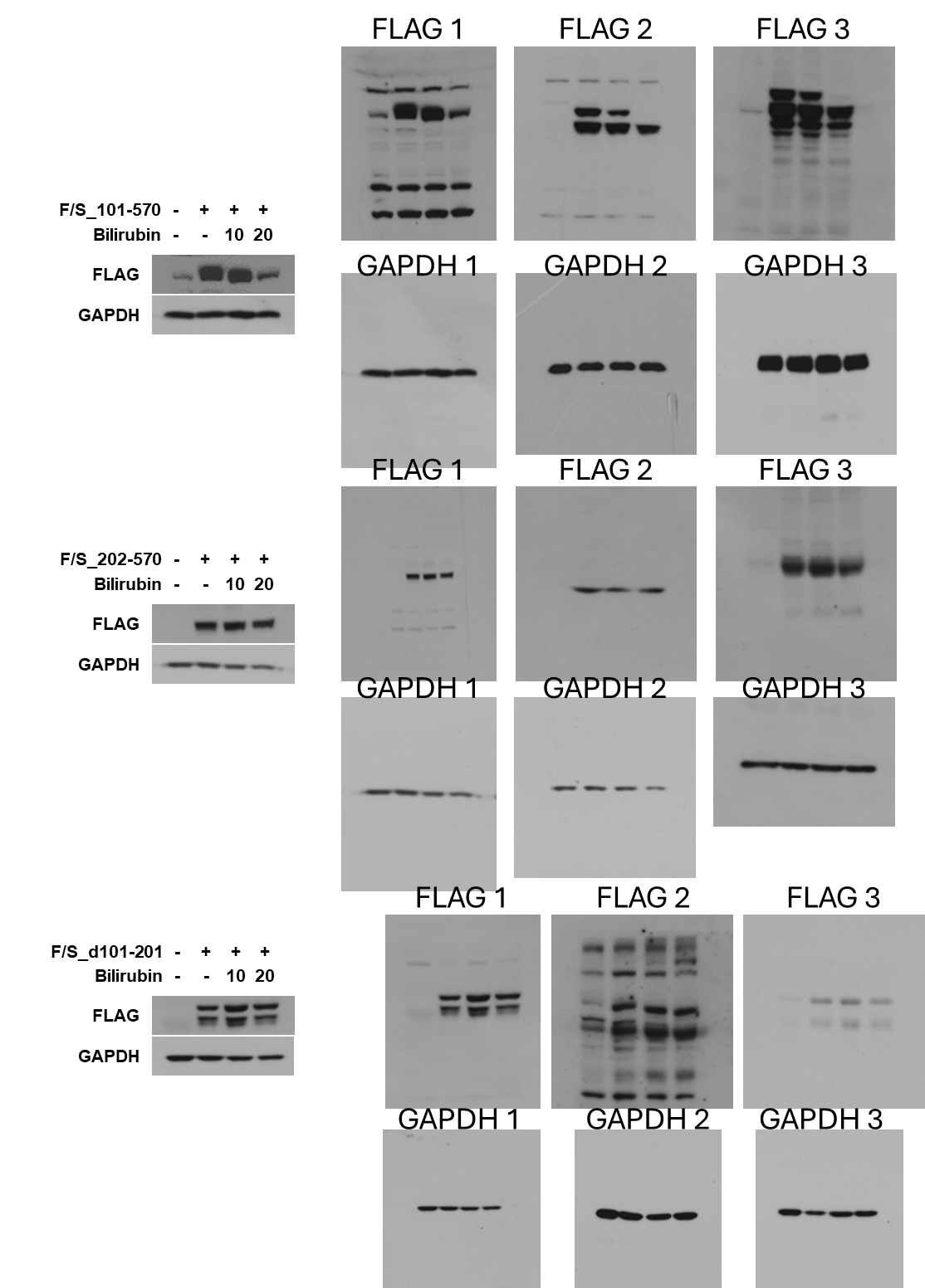


**Supplementary Figure S9. Uncropped Blots for Figure 6B**


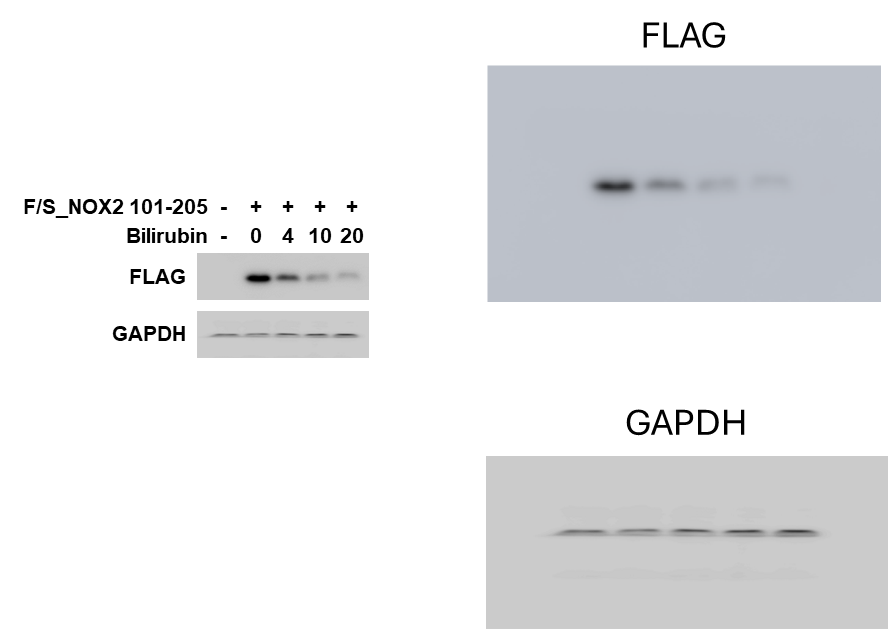


**Supplementary Figure S10. Uncropped Blots for Figure 6C**


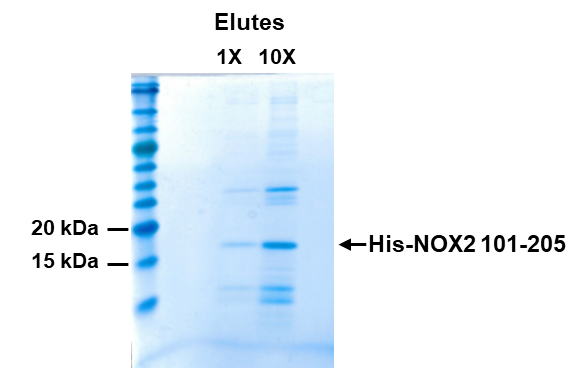


**Supplementary Figure S11. Purification of the NOX2 101–205 protein expressed by *E. coli.***

His-tagged NOX2 101–205 protein was expressed and purified from the soluble fraction of *E. coli* lysates. The His-tagged protein was induced with IPTG and purified using nickel-affinity chromatography. The proteins were eluted with imidazole from the nickel beads and the samples were concentrated about 10 times using an Amicon ultrafiltration membrane. To check the purity of the His-tagged protein, the proteins were subjected to SDS-PAGE, and visualized on the gel in EZ-gel staining solution.


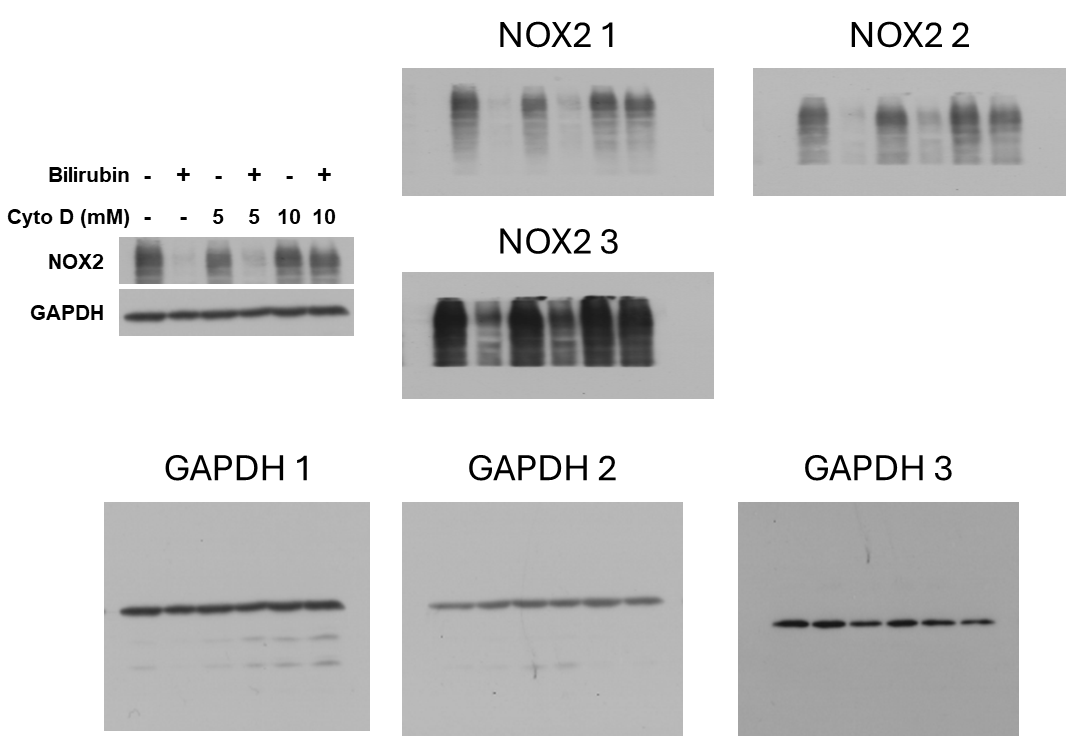


**Supplementary Figure S12. Uncropped Blots for Figure 8A**


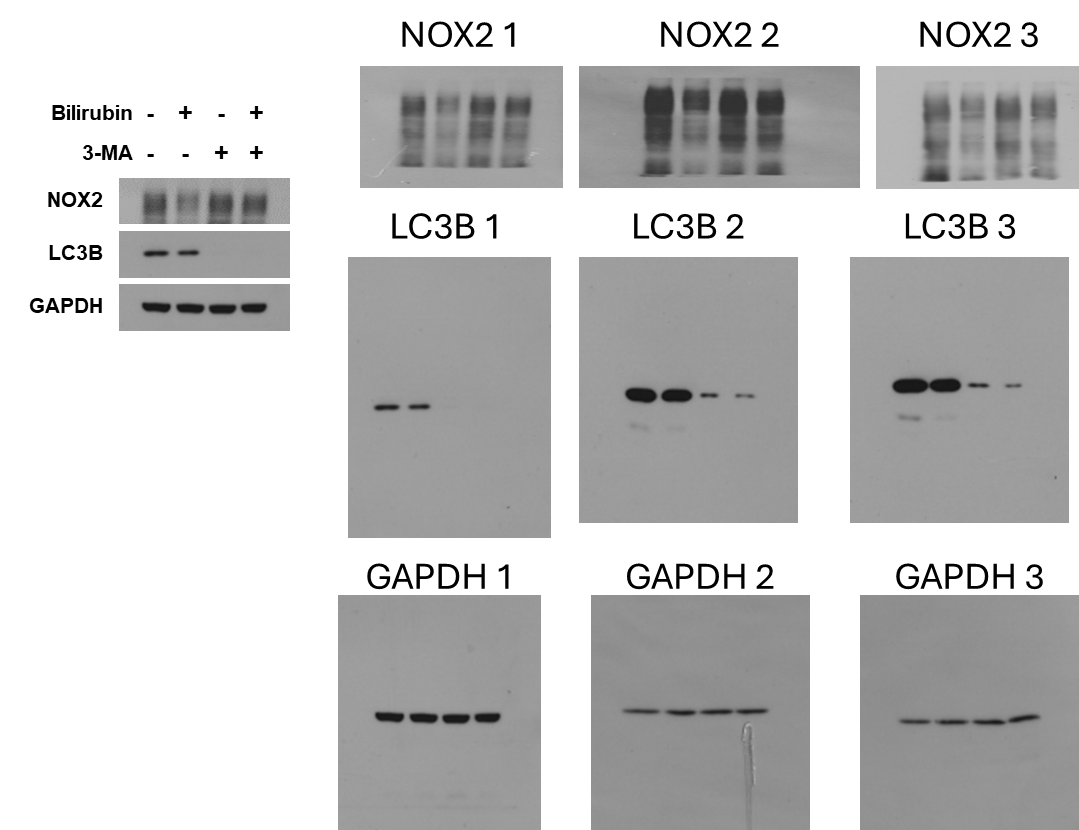


**Supplementary Figure S13. Uncropped Blots for Figure 8C**

| **Gene** | **primer** | **Sequence (5’ to 3’)** | **Accession code** |
| --- | --- | --- | --- |
| *GAPDH* | forward | GTCTCCTCTGACTTCAACAGCG | NM_002046 |
|  | reverse | ACCACCCTGTTGCTGTAGCCAA |  |
| *PDK4* | forward | AGGTGGAGCATTTCTCGCGCTA | NM_002612 |
|  | reverse | GAATGTTGGCGAGTCTCACAGG |  |
| *FABP1* | forward | AGAGCCAGGAAAACTTTGAA | NM_001443 |
|  | reverse | ACCACTGTCTTGACTTTCTC |  |
| *GK* | forward | ACTCAGTCATTTGACGGTAG | NM_000167 |
|  | reverse | TTCACTTTCCTCCGCATTAA |  |

**Supplementary Table 1.**
